# Supplementary material for: Detecting local genetic correlations with scan statistics
Source: Nat Commun. 2021 Apr 1;12:2033. doi: 10.1038/s41467-021-22334-6 (PMC8016883; doi:10.1038/s41467-021-22334-6)
Supplement: Supplementary file 1 — Supplementary Information [file 41467_2021_22334_MOESM1_ESM.pdf]

Supplementary Information

Detecting Local Genetic Correlations with Scan Statistics

Hanmin Guo, James J. Li, Qiongshi Lu, Lin Hou

Supplementary Notes

Supplementary Note 1. Functional form of scan statistic

We have proposed the scan statistic as follows, to search for regions harboring local genetic correlation:

$$Q(R) = \frac{\sum_{i\in R} z_{1i}z_{2i}}{(\sum_{i\in R} l_i)^{\theta}}.$$

As we mentioned in the main text, this is a generalization of the scan statistic proposed in Jeng et al[1] and Li et al[2], which detects signal regions associated with a single trait. The scan procedure in Jeng et al. was based on the mean of the marginal test statistics in a candidate region. We refer to this as the mean scan procedure. The scan statistic for a given region R is defined as $\frac{\sum_{i\in R} z_i}{\sqrt{|R|}}$, where $|R|$ denotes number of SNPs in region R. The authors showed that the mean scan procedure is asymptotically optimal as long as SNPs are independent and the SNPs in the signal region have the same effect size. In our paper, we extended the mean scan framework to the context of detecting local genetic correlation. We replace $|R| = \sum_{i\in R} 1$ in the mean scan procedure, with $\sum_{i\in R} l_i$, to account for LD. Notably, under the case that all the SNPs have the same LD effect, e.g. l_i are the same for all SNPs, $\sum_{i\in R} l_i$ just reduces to $|R|$ up to a multiple constant. Following Li et al[2], we relax the choice of power θ from 0.5 to a numeric in [0,1] to ensure that the scan statistic is comparable for different region sizes.

Second, the scan statistics we proposed has good genetics interpretation. It has been shown that under the polygenic model that per-SNP genetic covariance is the same for all SNPs, and if two GWASs are independent, then the following equation holds[3]:

$$E[z_{1i}z_{2i}] = \frac{\sqrt{N_1N_2}\rho_g}{M} l_i,$$

where the N_1, N_2 represents the sample size for two GWASs, M is the SNP count and ρ_g is the (global) genetic covariance. If we let θ be 1 and R be the whole genome, then the expectation of the scan statistic $Q(R)$ reduces to $\frac{\sqrt{N_1N_2}\rho_g}{M}$, which is exactly the (global) genetic covariance ρ_g multiplied by a constant. This implicates that when the candidate region is the whole genome, the scan statistic actually represents the strength of (global) genetic covariance. In our framework, we do not assume the polygenic model that per-SNP genetic covariance is the same for all SNPs across genome, but assume that genetic covariance is localized in some small genome regions. Therefore, we use the scan statistic in a local region, as a metric to detect significant local genetic sharing.

Supplementary Note 2. Simulation settings

Based on 503 individuals with European ancestry from the 1000 Genomes Project Phase 3 data[4], we simulated genotype data for 100,000 individuals with minor allele frequency (MAF) greater than 5% on chromosome 1 using HAPGEN2[5]. 336,532

variants remained in the dataset after removing strand ambiguous SNPs. Samples were randomly divided into two subsets with equal sample size, each with 50,000 individuals. We used each subset to simulate the phenotype data. We note that this genotype simulation approach is consistent with the simulation setting in the ρ-HESS paper[6].

First, we performed simulations under the null hypothesis. We simulated phenotype under different genetic architecture and evaluated whether our approach would produce false positive findings. Under the infinitesimal model, we assumed the effect size level of all the normalized SNPs are the same, and the per-normalized-SNP genetic effect was drawn from a normal distribution $\mathrm{N}(0, \frac{h^2}{336532})$ for both traits. Under a model realistic genetic architecture—heritability enrichment model, we attributed 30% of the trait heritability to 5000 randomly chosen SNPs, while the remaining SNPs explain 70% of the trait heritability. The per-SNP genetic effect was drawn from a normal distribution $\mathrm{N}(0, 0.3 * \frac{h^2}{5000})$ for SNPs with high heritability enrichment, and from $\mathrm{N}(0, 0.7 * \frac{h^2}{336532-5000})$ for SNPs with low heritability enrichment. To see whether there was false positive inflation with shared sample, we drew the effect size under the infinitesimal model as above, except that in this setting 25,000 samples were shared by two traits with environmental correlation 0.5. To study scenarios with different heritability, we fixed the heritability of the second trait at 0.02, and vary the heritability of the first trait. To simulate a binary trait, we first simulated the continuous liability following the infinitesimal model, then assigned the samples with liability greater than 50% quantile as cases and the rest as controls. We also considered scenarios with model mis-specification. Under the LDAK model[7] with MAF-dependent and LD-dependent effect sizes, we drew the genetic effect of the j-th SNP from a normal distribution $\mathrm{N}(0, h_j^2)$, where $h_j^2 \propto [f_j * (1 - f_j)]^{0.75} * w_j$, f_j is the minor allele frequency, and w_j is the weight computed by the LDAK software. Under the infinitesimal model with t-distributed effects, per-SNP heritability for each variant was set to be the same as before, but the effect size of each SNP is proportional to t-distribution with 10 degrees of freedom. Under the non-infinitesimal model with sparse effects, we split up chromosome 1 into two parts (i.e., chr1:1-116,000,000 and chr1:116,000,001-249,143,646), each containing half of the SNPs. For the first trait, we randomly sampled 10,000 variants from the first half of chr1 as causal variants. Similarly, we randomly sampled 10000 variants from the second half of chr1 as causal variants for the second trait. Per-SNP heritability for all the causal variants were the same as previous simulations. The trait heritability h^2 was set to vary from 0.01 to 0.05 in each scenario. Note that if we assume heritability is distributed proportionally in the genome, then the heritability values of 0.01 and 0.05 for chromosome 1 will correspond to the approximate heritability values of 0.12 and 0.60 for the whole

genome, respectively, which are on the same scale of SNP heritability for various traits. Each simulation setting was repeated for 100 times.

Next, we performed simulations to assess the statistical power. Under the heritability enrichment model, we randomly selected $N = 5$ segments, each containing $L = 1000$ SNPs, as the signal regions shared between two traits. We attributed $p = 0.3$ trait heritability to the signal regions. The genetic effect size for the SNPs in the signal regions follows a multivariate normal distribution

$$\begin{bmatrix}\beta_i \\ \gamma_i\end{bmatrix} \sim \mathrm{N}\left(\begin{bmatrix}0 \\ 0\end{bmatrix}, \begin{bmatrix}\frac{p * h^2}{NL} & \frac{p * h^2 * \rho}{NL} \\ \frac{p * h^2 * \rho}{NL} & \frac{p * h^2}{NL}\end{bmatrix}\right).$$

The genetic effect size for the SNPs outside the signal regions follows a different multivariate normal distribution without local genetic correlation

$$\begin{bmatrix}\beta_i \\ \gamma_i\end{bmatrix} \sim \mathrm{N}\left(\begin{bmatrix}0 \\ 0\end{bmatrix}, \begin{bmatrix}\frac{(1-p) * h^2}{336532 - NL} & 0 \\ 0 & \frac{(1-p) * h^2}{336532 - NL}\end{bmatrix}\right).$$

We evaluated the statistical power in different scenarios, with parameter h^2 varying from 0.01 to 0.05, the correlation of genetic effect size of two traits ρ varying from 0.2 to 0.8, proportion of heritability in signal regions p varying from 0.1 to 0.5, and length of signal region L varying from 250 to 2,000. To consider binary scenarios, we simulated two binary traits under the liability threshold model, where the continuous liabilities followed the same heritability enrichment model as previous simulations. We evaluated the statistical power in different scenarios, with total sample size fixed at 50,000 and sample prevalence varying from 0.01 to 0.5, and with effective sample size ($n_{eff} = \frac{4}{\frac{1}{n_{case}} + \frac{1}{n_{ctrl}}}$) fixed at 10,000 and sample prevalence varying from 0.1 to 0.5. In addition, we evaluated statistical power under mis-specified models. To model LD-dependent and MAF-dependent effect sizes, we simulated two phenotypes using the LDAK[7] framework. Under the non-infinitesimal model with sparse effects, we randomly sampled 10 causal regions for each trait, among which 5 causal regions were shared by both traits. Under the infinitesimal model with t-distributed effects, effect sizes for SNPs in signal regions were generated as follows:

$$\beta_i = \left(\sqrt{\rho}\xi_i + \sqrt{1-\rho}\zeta_i\right) * \sqrt{\frac{4ph^2}{5NL}}; \ \gamma_i = \left(\sqrt{\rho}\xi_i + \sqrt{1-\rho}\eta_i\right) * \sqrt{\frac{4ph^2}{5NL}},$$

and genetic effect sizes for the SNPs outside the signal regions were generated as follows:

$$\beta_i = \sqrt{\frac{4(1-p)h^2}{5(336532 - NL)}}\zeta_i; \ \gamma_i = \sqrt{\frac{4(1-p)h^2}{5(336532 - NL)}}\eta_i,$$

where ξ_i, ζ_i, η_i independently follow t_{10} distribution. The above genetic model guaranteed that the effect sizes are generated proportional to the t-distribution, and

the variance explained by all SNPs is exactly equal to the trait heritability, h^2. We varied the heritability h^2 as previous simulations. Each simulation setting was repeated for 100 times. Finally, we defined an identified region as false positive if its genome distance to the nearest true signal region is larger than 500 KB (to account for LD effect).

In simulation studies of ρ-HESS, we used the 133 approximately LD-independent regions[8] in chromosome 1 (1.6 Mb in width on average) as the pre-specified genomic regions, as recommended by the original paper of ρ-HESS[6].

We adjusted the significance cutoff of different approaches to achieve the same type I error. For coloc and gwas-pw, in those heritability settings with empirical type I error greater than 0.05, we increased the cutoff of the posterior probabilities so that the empirical type I error is controlled at 0.05.

Supplementary Note 3. Interpretation of genetic covariance enrichment

First, we provide a clear definition for genetic covariance enrichment. Suppose the genetic covariance between two traits explained by additive genetic effects is ρ_g, then the per-SNP genetic covariance is $\frac{\rho_g}{M}$ where M is the toal number of SNPs in the analysis. For a given genomic region (or multiple regions which we consider as a SNP set) containing m SNPs, we denote the local genetic covariance explained by SNPs within this genomic region as $\rho_{g,local}$, and the per-SNP genetic covariance within this genomic region is $\frac{\rho_{g,local}}{m}$. We define the genetic covariance enrichment of this given region to be $\frac{\rho_{g,local}}{m} / \frac{\rho_g}{M}$. This term quantifies the ratio of per-SNP heritability within the given genomic region and that in the genome. Such a definition is conceptually similar to the heritability enrichment widely used in the field[9] and can be quantified using tools such as GNOVA[10]. If a region shows strong enrichment for genetic covariance, it means that SNPs in this region have more contributions to the global genetic covariance compared to randomly selected regions in the genome.

In addition, to demonstrate that genetic covariance enrichment is a fair and effective metric for comparing different approaches, we performed two simulations (i.e. under the alternative and null) and evaluated the genetic covariance enrichment respectively. First, we simulated two genetically correlated traits and applied four methods (i.e., LOGODetect, ρ-HESS, gwas-pw, and coloc) to these two traits. We used 22 autosomes genotype data from the Wellcome Trust Case Control Consortium (WTCCC) cohort to perform simulations. We randomly selected $N = 100$ segments, each containing $L = 100$ SNPs, as the signal regions shared between two traits. We attributed $p = 0.3$ trait heritability to the signal regions. The effect size correlation of shared signal SNPs, ρ, is set to be 0.9. Heritability h^2 is set to be 0.5 for both traits. Under this setting, the genetic covariance enrichment is higher in the regions identified

by LOGODetect than regions identified by the other three methods (**Supplementary Figure 60 solid line**), which is concordant with the real data results between BIP and SCZ. To investigate whether the higher genetic covariance enrichment for the regions identified by LOGODetect are potential bias introduced by using LD scores in calculating the scan statistic, we performed null simulations. For each trait, we attributed 30% of the trait heritability to 10000 randomly chosen SNPs, while the remaining SNPs explain 70% of the trait heritability. We applied the four methods, LOGODetect, ρ-HESS, coloc, and gwas-pw to these simulated independent traits, and the corresponding top regions are identified. Then we evaluated the proportion of genetic covariance explained by these top regions with respect to the correlated traits under the alternative, and we found no significant enrichment (**Supplementary Figure 60 dashed line**). This demonstrates that LOGODetect does not favor regions with high LD scores and will not induce systematic bias in calculating genetic covariance enrichment. Thus, the observed enrichment of genetic covariance in real data analysis of LOGODetect regions is attributed to the shared genetic architecture instead of the systematic bias. We concluded that genetic covariance enrichment is a fair metric comparing performance of different methods, and LOGODetect actually identify more precise regions than other methods.

Supplementary Note 4. Replication of findings by LOGODetect

We applied LOGODetect to the UKBB summary statistics of BIP and SCZ, and investigated how many of 33 identified BIP-SCZ genomic regions could be replicated. However, In the UKBB, GWAS of BIP and SCZ had very limited case counts (n_{case}=1,064 for BIP; n_{case}=571 for SCZ) despite the large total sample size (n_{total}=366,540 for BIP; n_{total}=366,047 for SCZ), which led to insufficient statistical power for direct replication. We calculated the effective sample size, n_{eff}, for each study using the formula $n_{eff} = \frac{4}{\frac{1}{n_{case}}+\frac{1}{n_{ctrl}}} = 4n_{total}S(1-S)$, where S is the within-sample prevalence. n_{eff} for BIP and SCZ were 49,367 and 99,863 respectively in discovery GWASs, and only 4,244 and 2,280 in the UKBB.

In the following, we assessed the impact on statistical power given reduced n_{eff}. For simplicity, we treated all traits as continuous and assumed the total sample size in GWAS to be $n = n_{eff}$. Using z-score in GWAS summary statistics as input, we mimicked the down-sampled z-score analogous to the approach PUMAS[11] as $\mathbf{z}_0 = \sqrt{\frac{n_0}{n}}\mathbf{z} + \mathrm{N}\left(\mathbf{0}, \frac{n-n_0}{n}\mathbf{V}\right)$, where n_0 is the sample size of the random subsample and $\mathbf{V}$ is the LD (correlation) matrix and is approximated using empirical LD in a reference panel. Using this approach, we decreased the n_{eff} of SCZ and BIP GWASs to that in the UKBB replication cohort. As expected, the number of detected regions declined as n_{eff} decreased (**Supplementary Figure 61**). We do not expect to see any significant replication given the strength of local genetic correlation in input GWASs and sample size in the UKBB. Therefore, directly applying LOGODetect to the UKBB for replication would be statistically underpowered.

Instead of applying the direct replication approach, we chose to assess the enrichment of aggregated genetic covariance across all identified local genomic segments to replicate our findings. Here, we provided more empirical justifications to this approach. We repeated the down-sampling analysis described above but assessed the enrichment of genetic covariance in all identified regions in the replication dataset instead of statistical significance of each segment. We found that the detected regions are still expected to be substantially enriched for genetic covariance even when n_{eff} decreased to the level of UKBB replication cohort (**Supplementary Figure 62A**), whereas randomly selected regions show no enrichment for genetic covariance when n_{eff} varies (**Supplementary Figure 62B**).

In addition, in spite of the seven neuropsychiatric traits which we had investigated in the main text, we applied our method to two anthropometric traits: height and body-mass index (BMI), to see whether findings by LOGODetect can be replicated with a direct approach. We conducted analysis on summary statistics of height[12] (n=253,288) and BMI[13] (n=236,231) from the GIANT consortium and replicated our findings in the UKBB (n=455,332 and 454,841). We identified 14 regions with significant local genetic correlation in the discovery analysis. 10 of 14 regions identified in the discovery stage were successfully replicated, suggesting the effectiveness of LOGODetect to identify replicable genomic regions with local genetic correlations.

Supplementary Note 5. $|\sum_{i\in R} z_{1i} z_{2i}|$ is larger in regions with strong LD

We observed the pattern that $|\sum_{i\in R} z_{1i} z_{2i}|$ is larger in regions with strong LD in real GWASs. We used BIP and SCZ as an example. We partitioned the genome into 30,957 blocks, each spanning 200 SNPs. Then we grouped these blocks into three equally-sized categories (i.e., high LD, medium LD, and low LD) according to their LD strength (sum of LD scores of SNPs in each block). Each category contains 10,319 blocks. Two sample t-tests for each category pair suggest significantly larger $|\sum_{i\in R} z_{1i} z_{2i}|$ in blocks with strong LD (maximum p=2.16e-68) (**Supplementary Figure 59**).
And
We also used a toy example to illustrate the observation that expected absolute value of $\sum_{i\in R} z_{1i} z_{2i}$ is larger in regions with strong LD. Consider two independent GWAS with K causal SNPs: $\mathbf{y}_1 = \sum_{i=1}^{K} \mathbf{X}_i^T \beta_i + \boldsymbol{\epsilon}, \mathbf{y}_2 = \sum_{i=1}^{K} \mathbf{Z}_i^T \gamma_i + \boldsymbol{\delta}$, where $\mathbf{y}_1, \mathbf{y}_2$ are standardized trait vectors with n_1, n_2 samples respectively, $\mathbf{X}_i, \mathbf{Z}_i$ are standardized genotypes of i-th SNP, $\boldsymbol{\epsilon}$ and $\boldsymbol{\delta}$ are independent environment effect. Suppose $\begin{bmatrix}\boldsymbol{\beta}\\ \boldsymbol{\gamma}\end{bmatrix} \sim \mathrm{N}\left(\begin{bmatrix}\mathbf{0}\\ \mathbf{0}\end{bmatrix}, \begin{bmatrix}\frac{0.1}{K}\mathbf{I}_K & \frac{0.05}{K}\mathbf{I}_K\\ \frac{0.05}{K}\mathbf{I}_K & \frac{0.1}{K}\mathbf{I}_K\end{bmatrix}\right)$, and suppose the first $K-1$ SNPs are in perfect LD, and are in perfect LE (linkage equilibrium) with the K-th SNP, which means $\mathbf{X}_1 = \mathbf{X}_2 = \cdots = \mathbf{X}_{K-1}, \mathbf{Z}_1 = \mathbf{Z}_2 = \cdots = \mathbf{Z}_{K-1}$, and $\mathbf{X}_1^T \mathbf{X}_K = \mathbf{Z}_1^T \mathbf{Z}_K = 0$. Suppose the environment effect is independent with the genotype. We ignore the distinction between independence in population level and in sample level, which means $\mathbf{X}_i^T \boldsymbol{\epsilon} = 0, \mathbf{Z}_i^T \boldsymbol{\delta} =$

$0, \boldsymbol{\epsilon}^T \boldsymbol{\delta} = 0$. Note that $z_{1i} = \frac{1}{\sqrt{n_1}} \mathbf{X}_i^T \mathbf{y}_1, z_{2i} = \frac{1}{\sqrt{n_2}} \mathbf{Z}_i^T \mathbf{y}_2$, it can be shown that,

$$\left|\sum_{i=1}^{K-1} z_{1i} z_{2i}\right| = (K-1)|z_{11} z_{21}|$$

$$= \frac{K-1}{\sqrt{n_1 n_2}} \left|\mathbf{X}_1^T \left(\mathbf{X}_1 \sum_{i=1}^{K-1} \beta_i + \mathbf{X}_K \beta_K + \boldsymbol{\epsilon}\right) * \mathbf{Z}_1^T \left(\mathbf{Z}_1 \sum_{i=1}^{K-1} \gamma_i + \mathbf{Z}_K \gamma_K + \boldsymbol{\delta}\right)\right|$$

$$= \frac{K-1}{\sqrt{n_1 n_2}} \left|n_1 \sum_{i=1}^{K-1} \beta_i * n_2 \sum_{i=1}^{K-1} \gamma_i\right|$$

$$= (K-1)\sqrt{n_1 n_2} \left|\sum_{i=1}^{K-1} \beta_i \gamma_i\right|.$$

Similarly we have $\left|\sum_{i=K}^{K} z_{1i} z_{2i}\right| = \sqrt{n_1 n_2} |\beta_K \gamma_K|$. Therefore, inequality $E\left|\sum_{i=1}^{K-1} z_{1i} z_{2i}\right| \geq E\left|\sum_{i=K}^{K} z_{1i} z_{2i}\right|$ holds if $E\left|\sum_{i=1}^{K-1} \beta_i \gamma_i\right| \geq E|\beta_K \gamma_K|$. To prove the latter inequality, one only needs to show that if $\xi_1, \xi_2 \ldots \xi_K$ are independently and identically distributed random variables, then $E\left|\sum_{i=1}^{K-1} \xi_i\right| \geq E|\xi_K|$. Suppose the cumulative distribution function of ξ_i is denoted by F, then $E|\xi_1 + \xi_2| - E|\xi_1 - \xi_2| = \int_0^\infty [1 - F(u) - F(-u)]^2 du \geq 0$. Thus $E|\xi_1 + \xi_2| \geq \frac{1}{2}[E|\xi_1 + \xi_2| + E|\xi_1 - \xi_2|] \geq E|\xi_1| = E|\xi_K|$. Similarly, one can show that $E\left|\sum_{i=1}^{K-1} \xi_i\right| \geq E|\xi_K|$, and it follows that $E\left|\sum_{i=1}^{K-1} z_{1i} z_{2i}\right| \geq E\left|\sum_{i=K}^{K} z_{1i} z_{2i}\right|$. This indicates that the expected absolute value of $\sum_{i \in R} z_{1i} z_{2i}$ is larger in regions with strong LD.

Supplementary Figures

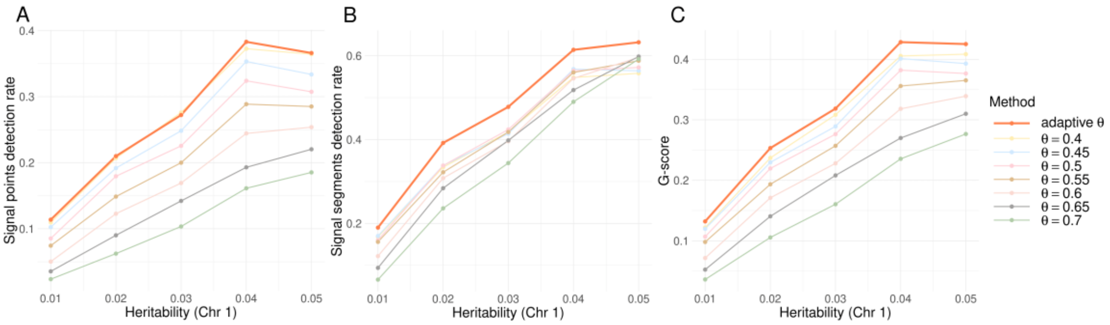

Supplementary Figure 1. Assessment of statistical power under a heritability enrichment model with varying trait heritability. (A-C) show statistical power assessed by three measures: Signal points detection rate, Signal segments detection rate and G-score. Heritability represents the trait heritability for both traits on chromosome 1. For each trait, we randomly choose N=5 segments, each containing L=1000 SNPs, as the signal regions. The heritability for the signal regions is set to be 30% of trait heritability. The correlation of genetic effect size of two traits ρ is set to be 0.9. Each simulation setting was repeated 100 times.

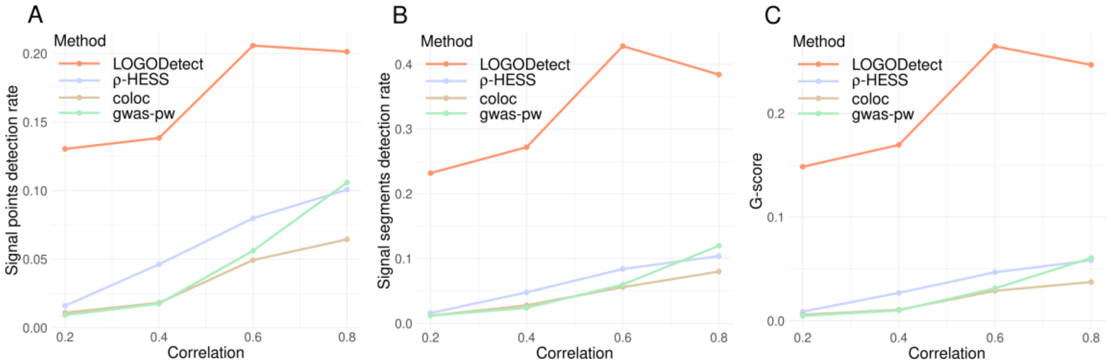

Supplementary Figure 2. Assessment of statistical power for four methods under a heritability enrichment model with varying correlation. (A-C) show statistical power assessed by three measures: Signal points detection rate, Signal segments detection rate and G-score. Correlation represents the correlation of genetic effect size of two traits. For each trait, we randomly choose N=5 segments, each containing L=1000 SNPs, as the signal regions. The heritability for the signal regions is set to be 30% of trait heritability. The trait heritability for two traits is set to be 0.03. Each simulation setting is repeated for 100 times. Here, significance cutoffs for coloc and gwas-pw were adjusted so that the empirical type I error rate for each method is controlled at 0.05.

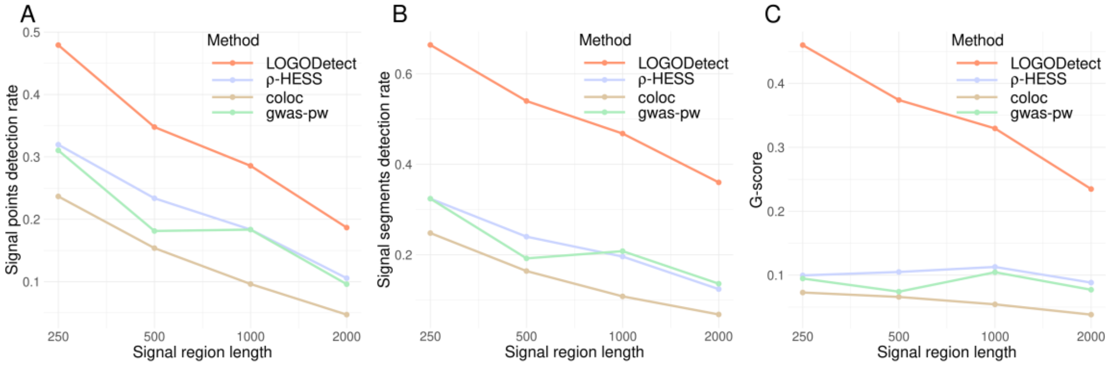

Supplementary Figure 3. Assessment of statistical power for four methods under a heritability enrichment model with varying signal region length. (A-C) show statistical power assessed by three measures: Signal points detection rate, Signal segments detection rate and G-score. Horizontal axis is in log scale. Signal region length denoted as L, represents the length of one true signal region. For each trait, we randomly choose N=5 segments, each containing L SNPs, as the signal regions. The trait heritability is set to be 0.03 for both traits. The heritability for the signal regions is set to be 30% of trait heritability. The correlation of genetic effect size of two traits ρ is set to be 0.9. Each simulation setting is repeated for 100 times. Here, significance cutoffs for coloc and gwas-pw were adjusted so that the empirical type I error rate for each method is controlled at 0.05.

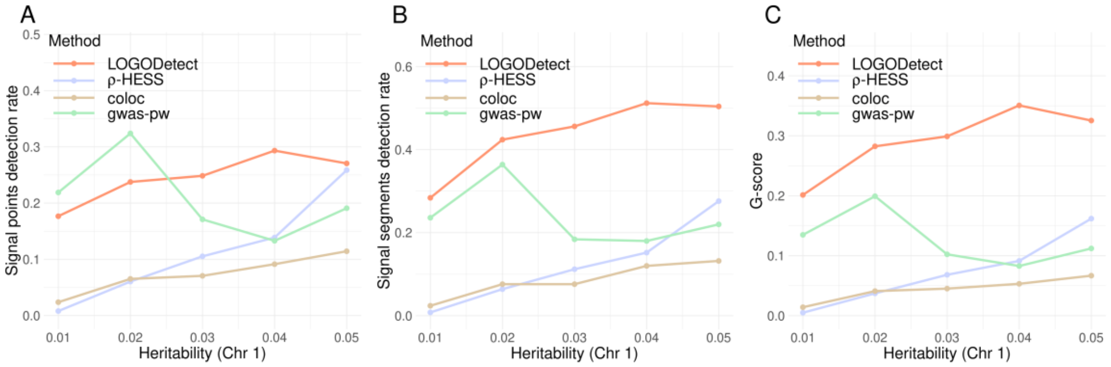

Supplementary Figure 4. Assessment of statistical power for four methods under a heritability enrichment model with varying heritability for the first trait and fixed heritability for the second trait. (A-C) show statistical power assessed by three measures: Signal points detection rate, Signal segments detection rate and G-score. Heritability represents the trait heritability for the first trait. The trait heritability for the second trait is set to be 0.02. For each trait, we randomly choose N=5 segments, each containing L=1000 SNPs, as the signal regions. The heritability for the signal regions is set to be 30% of trait heritability. The correlation of genetic effect size of two traits ρ is set to be 0.9. Each simulation setting is repeated for 100 times. Here, significance cutoffs for coloc and gwas-pw were adjusted so that the empirical type I error rate for each method is controlled at 0.05.

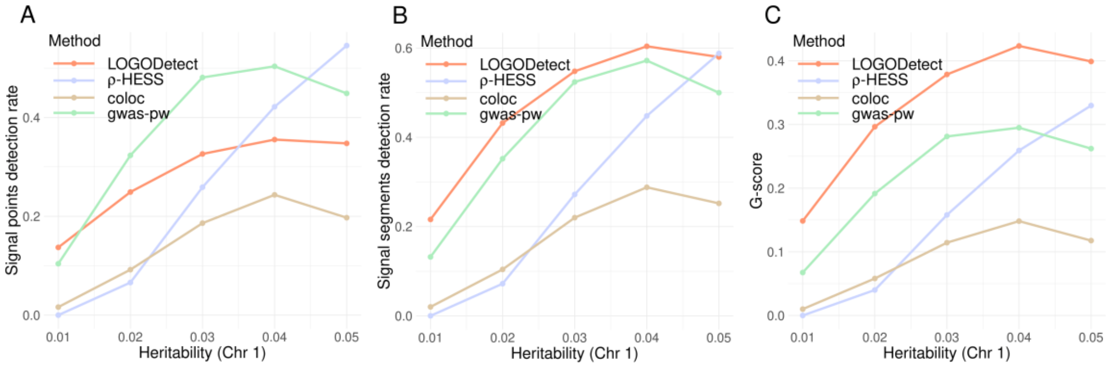

Supplementary Figure 5. Assessment of statistical power for four methods under a heritability enrichment model with 50% shared samples. (A-C) show statistical power assessed by three measures: Signal points detection rate, Signal segments detection rate and G-score. Heritability represents the trait heritability for both traits. For each trait, we randomly choose N=5 segments, each containing L=1000 SNPs, as the signal regions. The heritability for the signal regions is set to be 30% of trait heritability. The correlation of genetic effect size of two traits ρ is set to be 0.9. Each simulation setting is repeated for 100 times. Here, significance cutoffs for coloc and gwas-pw were adjusted so that the empirical type I error rate for each method is controlled at 0.05.

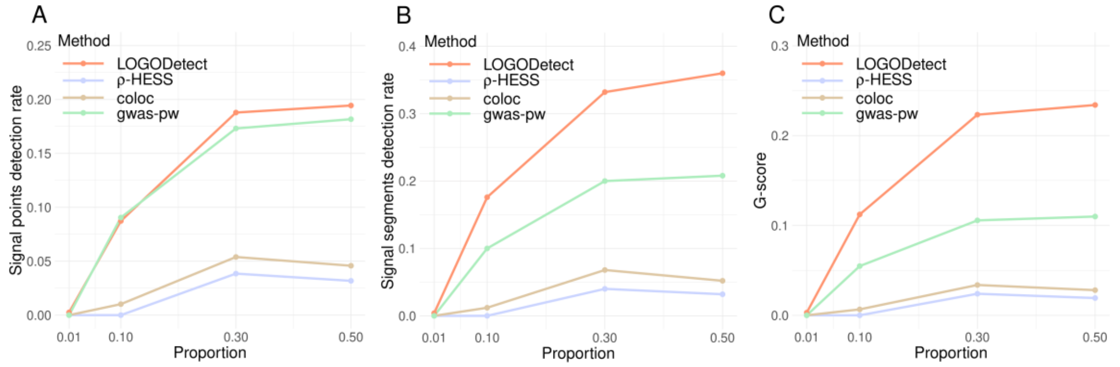

Supplementary Figure 6. Assessment of statistical power for four methods under a liability threshold model with varying sample prevalence and fixed total sample size. (A-C) show statistical power assessed by three measures: Signal points detection rate, Signal segments detection rate and G-score. Here we assume the liability threshold model that two binary traits are determined by unobserved continuous liabilities, where the liabilities follow the same heritability enrichment model in previous simulations. Proportion represents the sample prevalence (the proportion of cases in whole samples). Population prevalence and sample prevalence were set to be the same, varying from 0.01 to 0.5. Total sample size is fixed at 50,000 for each trait. For each trait, we randomly choose N=5 segments, each containing L=1000 SNPs, as the signal regions. The trait heritability is set to be 0.03 for both traits. The heritability for the signal regions is set to be 30% of trait heritability. The correlation of genetic effect size of two traits ρ is set to be 0.9. Each simulation setting is repeated for 100 times. Here, significance cutoffs for coloc and gwas-pw were adjusted so that the empirical type I error rate for each method is controlled at 0.05.

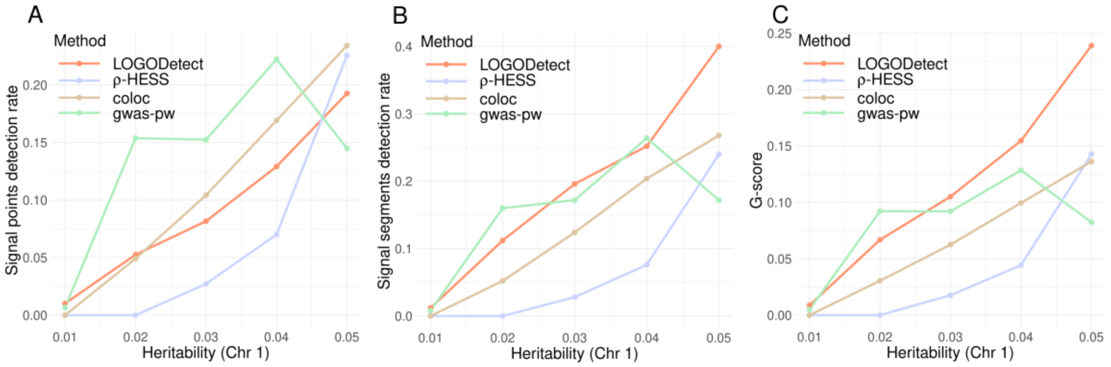

Supplementary Figure 7. Assessment of statistical power for four methods under a LDAK model. (A-C) show statistical power assessed by three measures: Signal points detection rate, Signal segments detection rate and G-score. Heritability represents the trait heritability for both traits. For each trait, we randomly choose N=5 segments, each containing L=1000 SNPs, as the signal regions. Here we assume a LDAK genetic architecture, where the j-th SNP heritability is proportional to $[f_j(1-f_j)]^{0.75} * w_j$, f_j and w_j denote minor allele frequency and weight computed by the LDAK software, respectively. The heritability for the signal regions is set to be 30% of trait heritability. The correlation of genetic effect size of two traits ρ is set to be 0.9. Each simulation setting is repeated for 100 times. Here, significance cutoffs for coloc and gwas-pw were adjusted so that the empirical type I error rate for each method is controlled at 0.05.

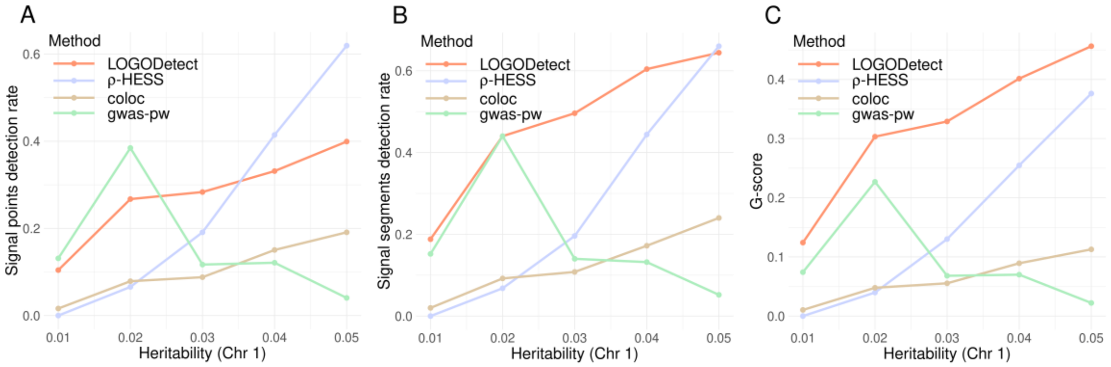

Supplementary Figure 9. Assessment of statistical power for four methods under an infinitesimal model with t-distributed effects. (A-C) show statistical power assessed by three measures: Signal points detection rate, Signal segments detection rate and G-score. Here we assume the infinitesimal model with t-distributed effects. For each trait, we randomly choose N=5 segments, each containing L=100 SNPs, as the signal regions. The heritability for the signal regions is set to be 30% of trait heritability. The correlation of genetic effect size of two traits ρ is set to be 0.9. Each simulation setting is repeated for 100 times. Here, significance cutoffs for coloc and gwas-pw were adjusted so that the empirical type I error rate for each method is controlled at 0.05.

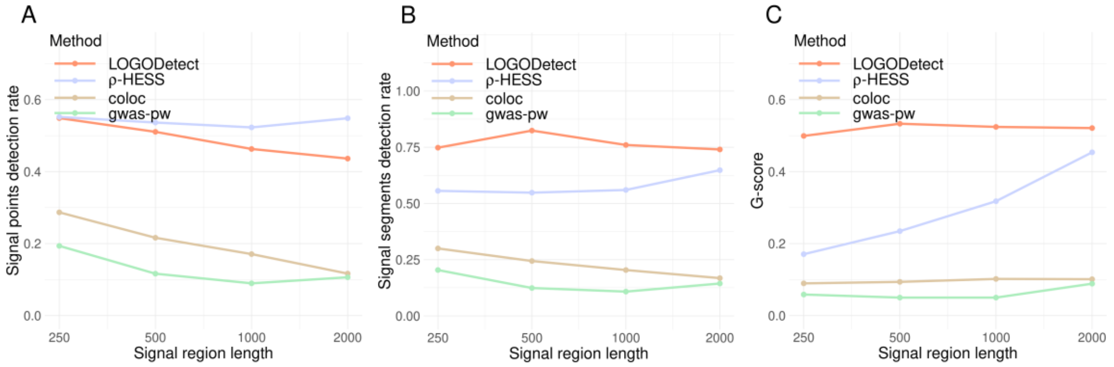

Supplementary Figure 10. Assessment of statistical power for four methods under a non-infinitesimal model with sparse effects. (A-C) show statistical power assessed by three measures: Signal points detection rate, Signal segments detection rate and G-score. Horizontal axis is in log scale. Signal region length, denoted as L, represents the length of one true signal region. Here we assume a sparse genetic model with few causal SNPs. We randomly sampled 10 causal regions for each trait, among which 5 causal regions were shared by both traits. Each causal region contains L SNPs. We assumed the per-SNP heritability for all the causal variants to be the same. The trait heritability is set to be 0.03 for both traits. The heritability for the signal regions is set to be 30% of trait heritability. The effect size correlation of shared causal regions, ρ, is set to be 0.9. Each simulation setting is repeated for 100 times. Here, significance cutoffs for coloc and gwas-pw were adjusted so that the empirical type I error rate for each method is controlled at 0.05.

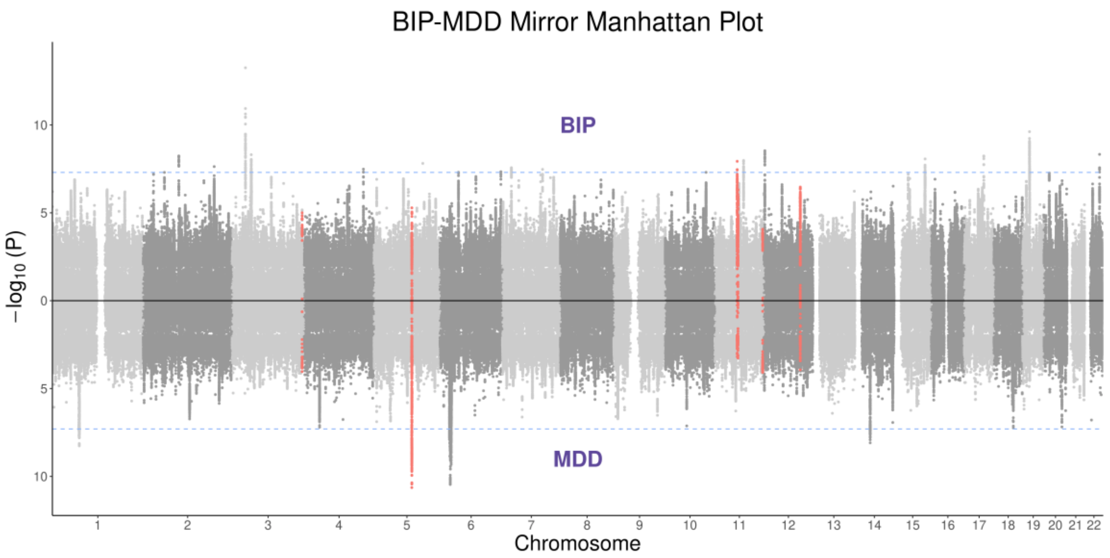

Supplementary Figure 11. Mirror Manhattan plot for BIP-MDD. Red dots represent SNPs located in the LOGODetect detected regions.

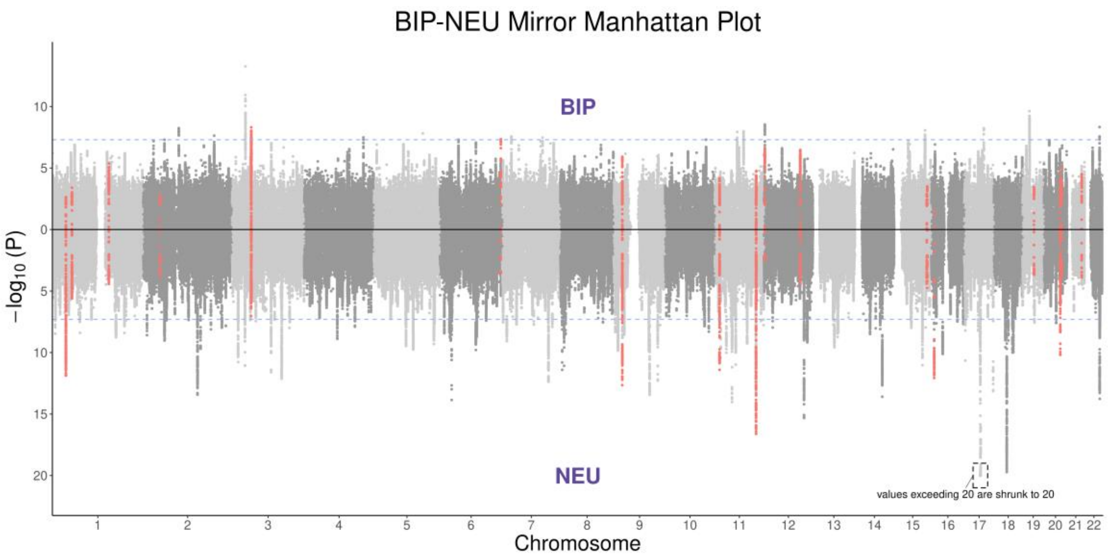

Supplementary Figure 12. Mirror Manhattan plot for BIP-NEU. Red dots represent SNPs located in the LOGODetect detected regions. For NEU, one locus on chromosome 17 have $-\log_{10} P$ value exceeding 20, those values are shrunk to 20 for conciseness.

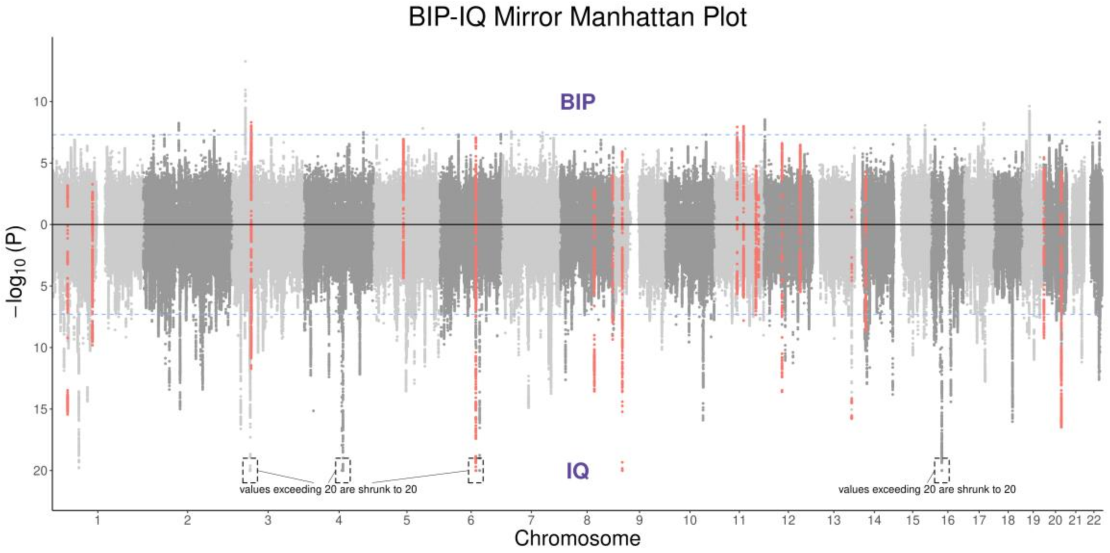

Supplementary Figure 13. Mirror Manhattan plot for BIP-IQ. Red dots represent SNPs located in the LOGODetect detected regions. For IQ, four loci on chromosome 3, 4, 6, and 16 have $-\log_{10} P$ value exceeding 20, those values are shrunk to 20 for conciseness.

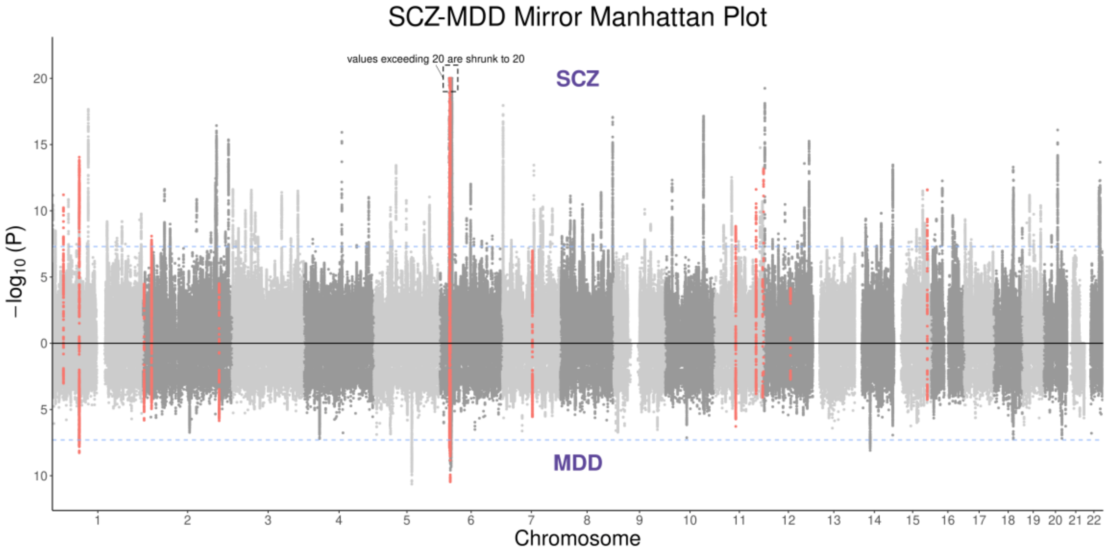

Supplementary Figure 14. Mirror Manhattan plot for SCZ-MDD. Red dots represent SNPs located in the LOGODetect detected regions. For SCZ, one locus on chromosome 6 have $-\log_{10} P$ value exceeding 20, those values are shrunk to 20 for conciseness.

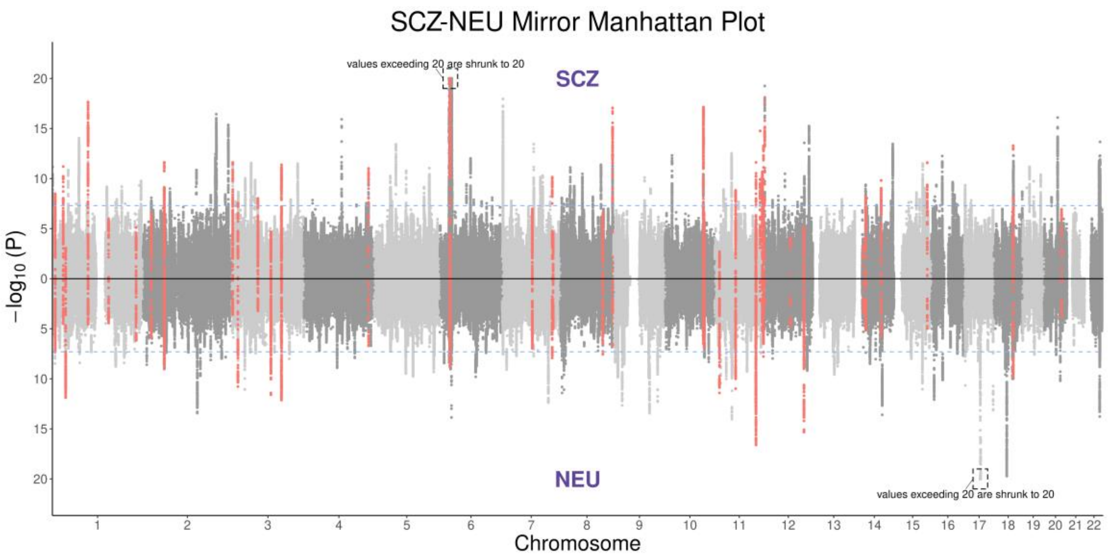

Supplementary Figure 15. Mirror Manhattan plot for SCZ-NEU. Red dots represent SNPs located in the LOGODetect detected regions. For SCZ, one locus on chromosome 6 have $-\log_{10} P$ value exceeding 20, for NEU, one locus on chromosome 17 have $-\log_{10} P$ value exceeding 20, those values are shrunk to 20 for conciseness.

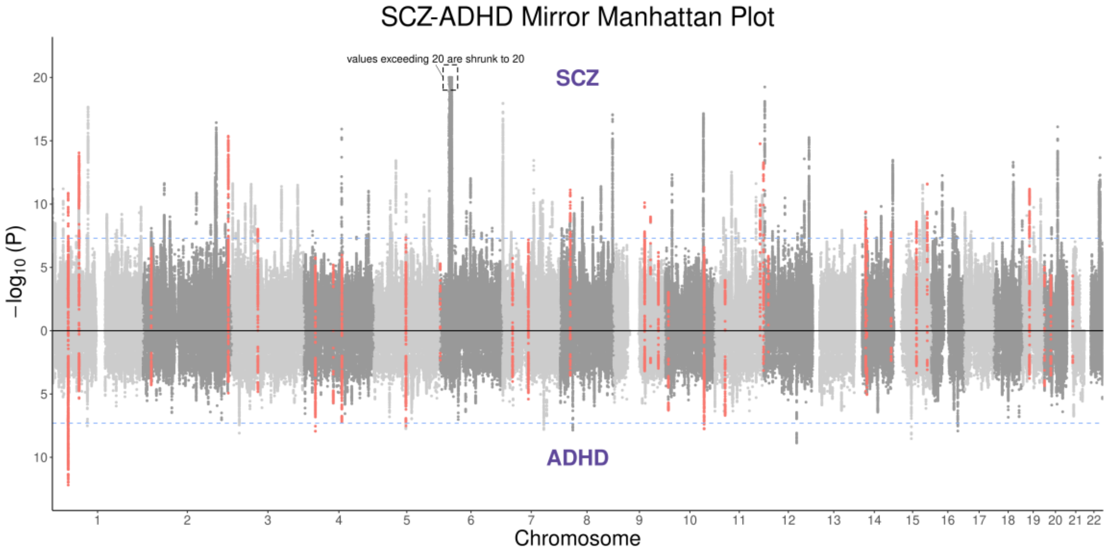

Supplementary Figure 16. Mirror Manhattan plot for SCZ-ADHD. Red dots represent SNPs located in the LOGODetect detected regions. For SCZ, one locus on chromosome 6 have $-\log_{10} P$ value exceeding 20, those values are shrunk to 20 for conciseness.

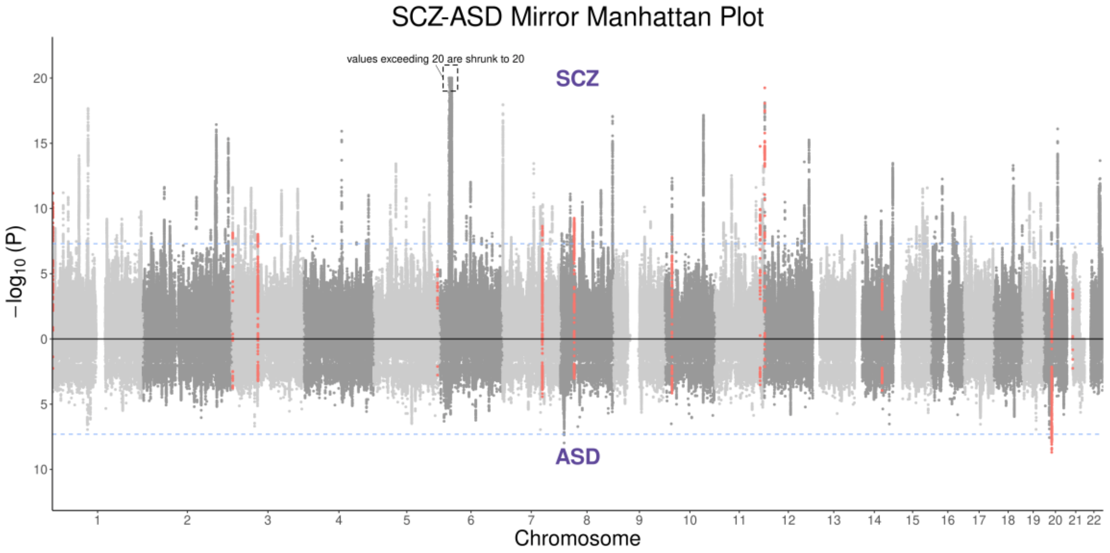

Supplementary Figure 17. Mirror Manhattan plot for SCZ-ASD. Red dots represent SNPs located in the LOGODetect detected regions. For SCZ, one locus on chromosome 6 have $-\log_{10} P$ value exceeding 20, those values are shrunk to 20 for conciseness.

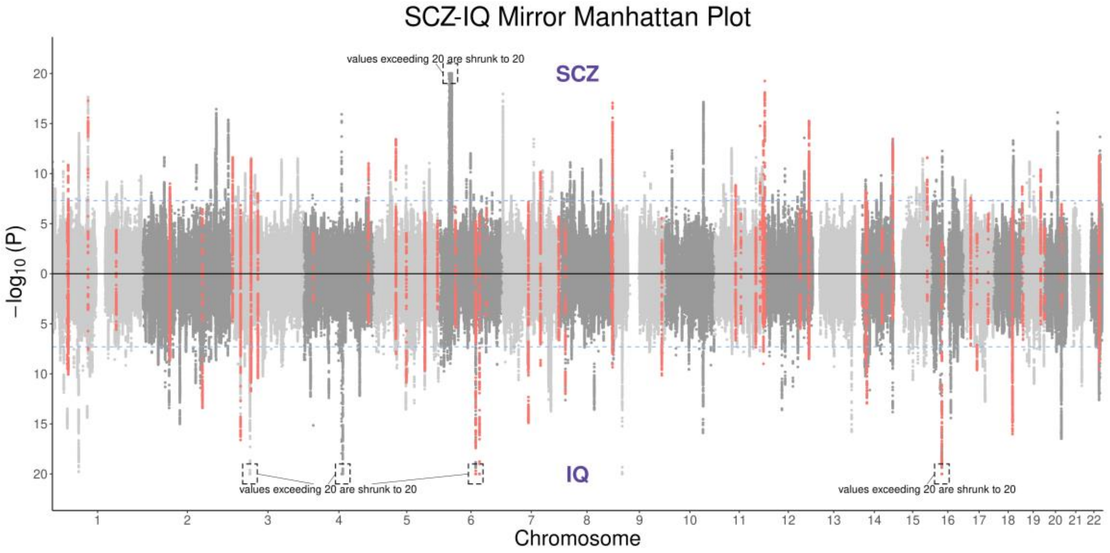

Supplementary Figure 18. Mirror Manhattan plot for SCZ-IQ. Red dots represent SNPs located in the LOGODetect detected regions. For SCZ, one locus on chromosome 6 have $-\log_{10} P$ value exceeding 20, for IQ, four loci on chromosome 3, 4, 6, and 16 have $-\log_{10} P$ value exceeding 20, those values are shrunk to 20 for conciseness.

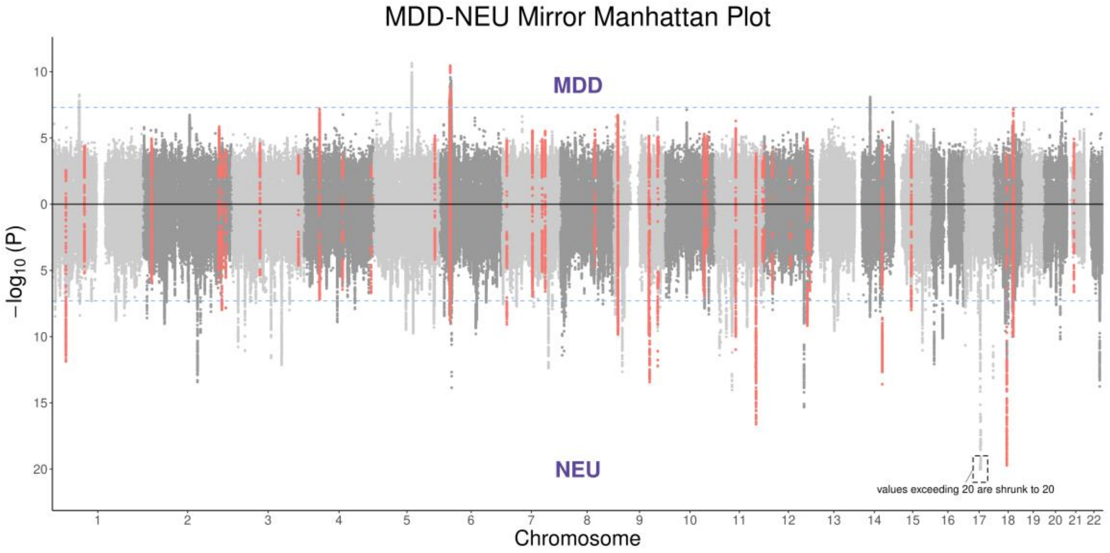

Supplementary Figure 19. Mirror Manhattan plot for MDD-NEU. Red dots represent SNPs located in the LOGODetect detected regions. For NEU, one locus on chromosome 17 have $-\log_{10} P$ value exceeding 20, those values are shrunk to 20 for conciseness.

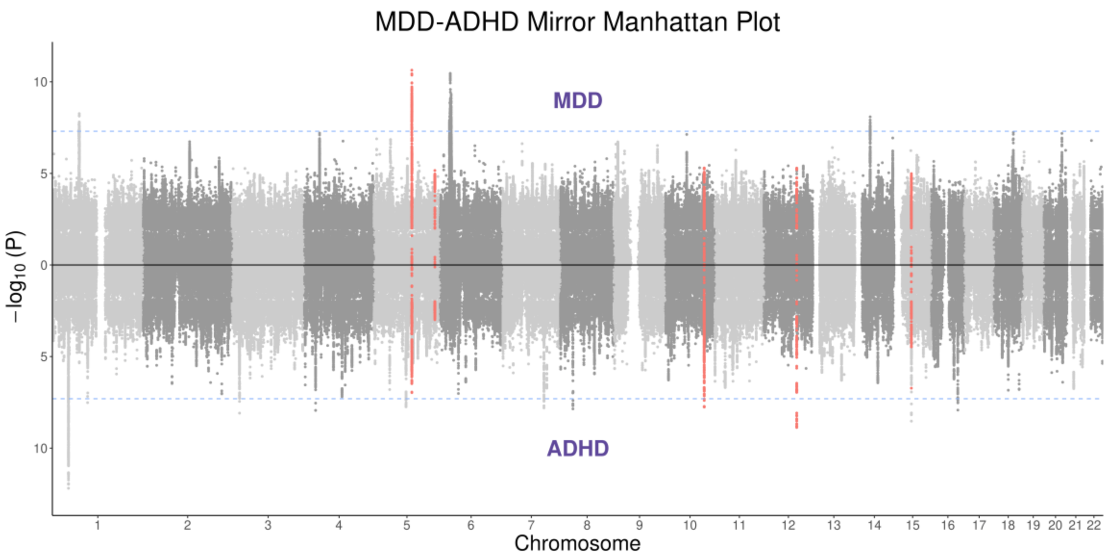

Supplementary Figure 20. Mirror Manhattan plot for MDD-ADHD. Red dots represent SNPs located in the LOGODetect detected regions.

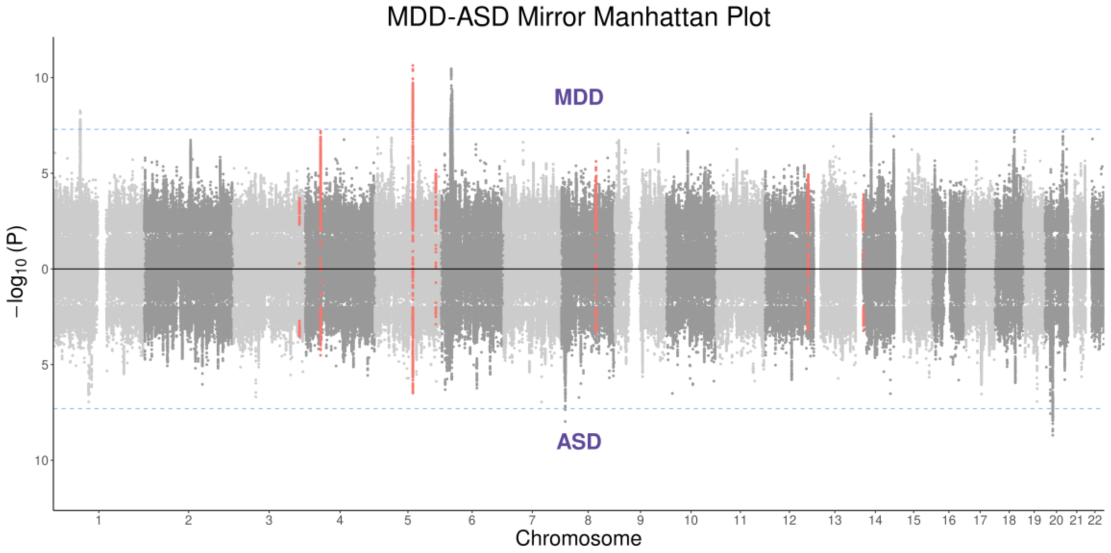

Supplementary Figure 21. Mirror Manhattan plot for MDD-ASD. Red dots represent SNPs located in the LOGODetect detected regions.

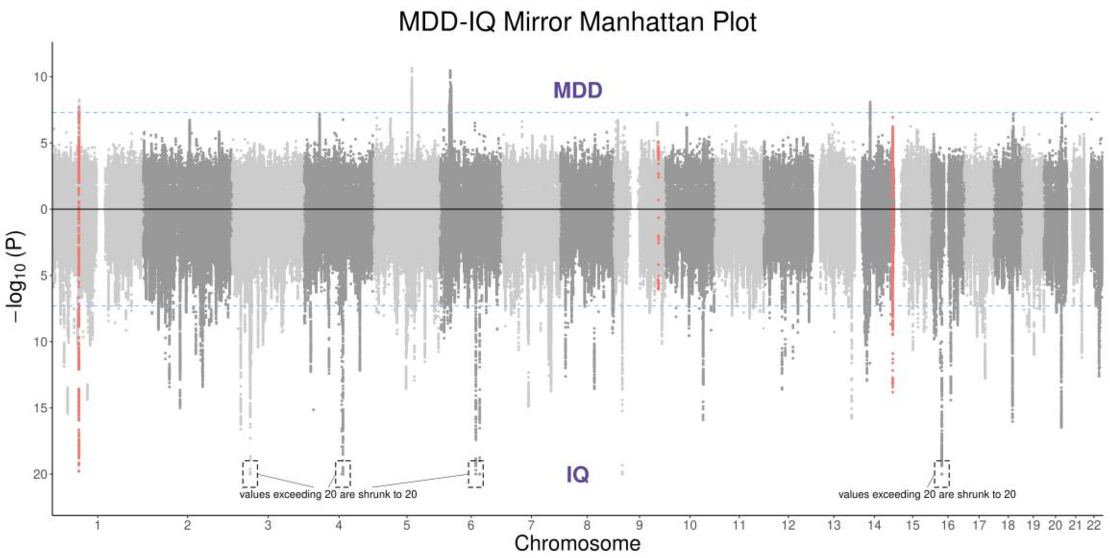

Supplementary Figure 22. Mirror Manhattan plot for MDD-IQ. Red dots represent SNPs located in the LOGODetect detected regions. For IQ, four loci on chromosome 3, 4, 6, and 16 have $-\log_{10} P$ value exceeding 20, those values are shrunk to 20 for conciseness.

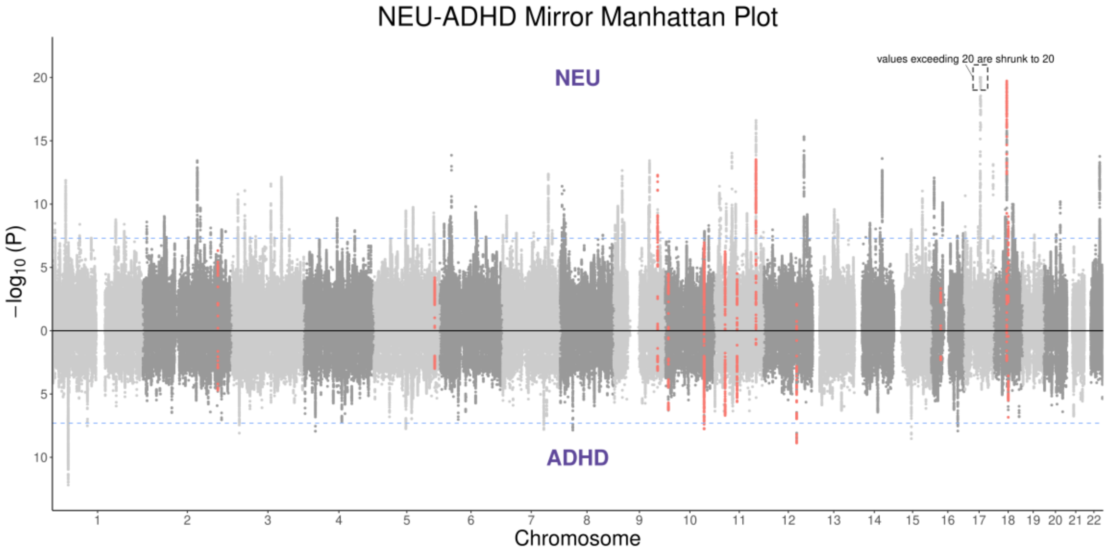

Supplementary Figure 23. Mirror Manhattan plot for NEU-ADHD. Red dots represent SNPs located in the LOGODetect detected regions. For NEU, one locus on chromosome 17 have $-\log_{10} P$ value exceeding 20, those values are shrunk to 20 for conciseness.

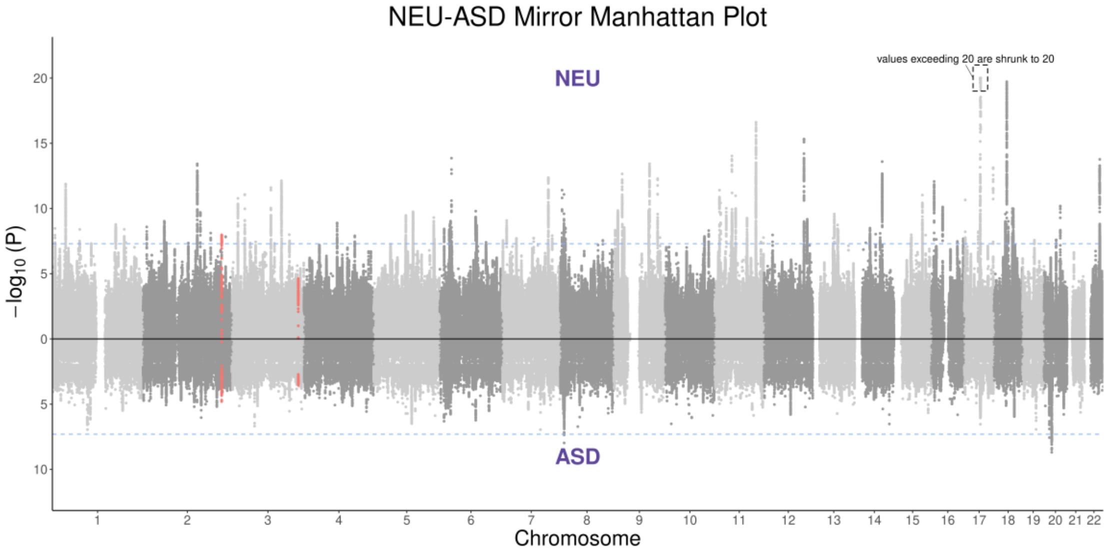

Supplementary Figure 24. Mirror Manhattan plot for NEU-ASD. Red dots represent SNPs located in the LOGODetect detected regions. For NEU, one locus on chromosome 17 have $-\log_{10} P$ value exceeding 20, those values are shrunk to 20 for conciseness.

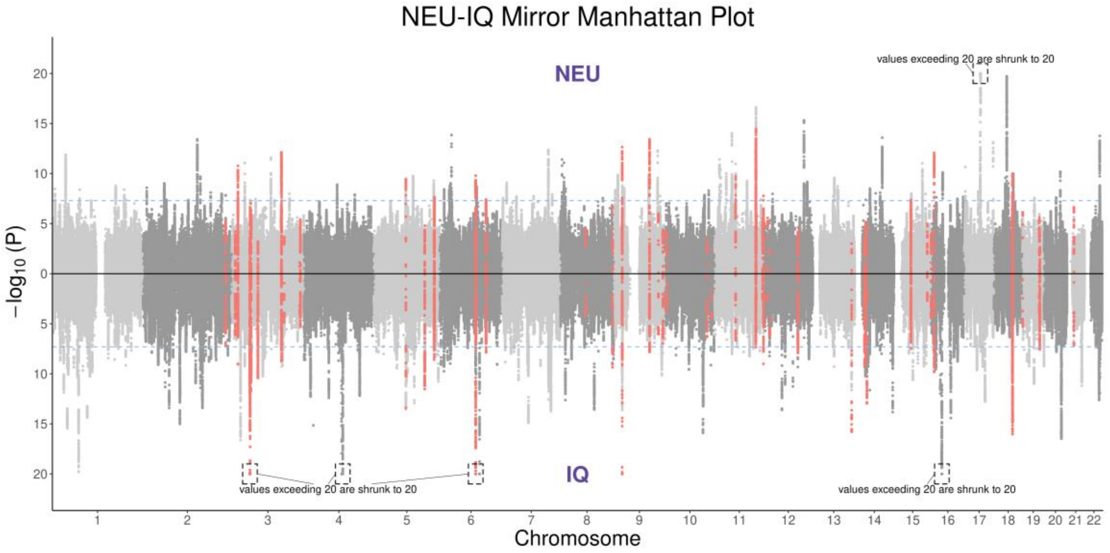

Supplementary Figure 25. Mirror Manhattan plot for NEU-IQ. Red dots represent SNPs located in the LOGODetect detected regions. For NEU, one locus on chromosome 17 have $-\log_{10} P$ value exceeding 20, for IQ, four loci on chromosome 3, 4, 6, and 16 have $-\log_{10} P$ value exceeding 20, those values are shrunk to 20 for conciseness.

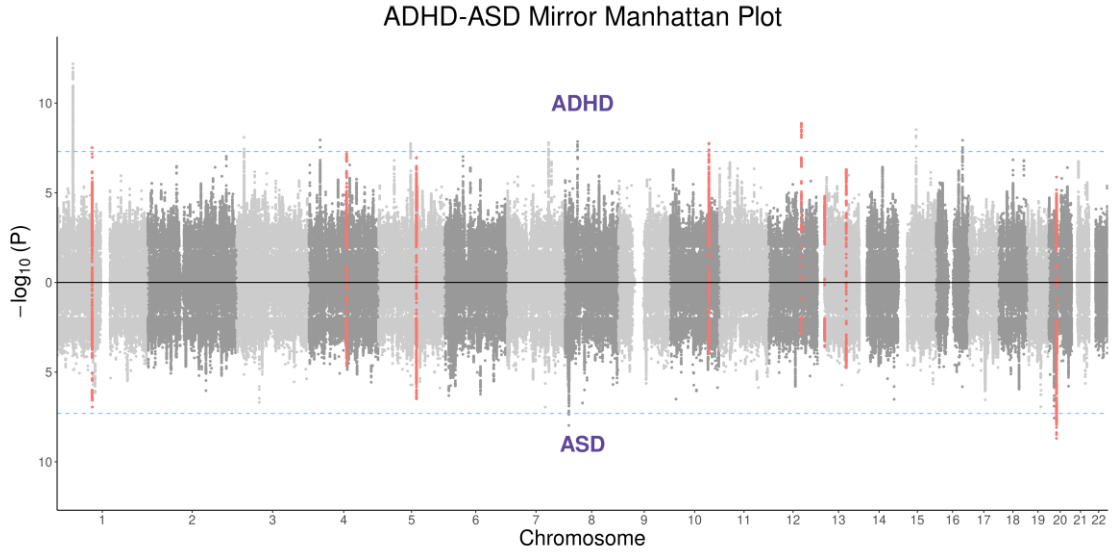

Supplementary Figure 26. Mirror Manhattan plot for ADHD-ASD. Red dots represent SNPs located in the LOGODetect detected regions.

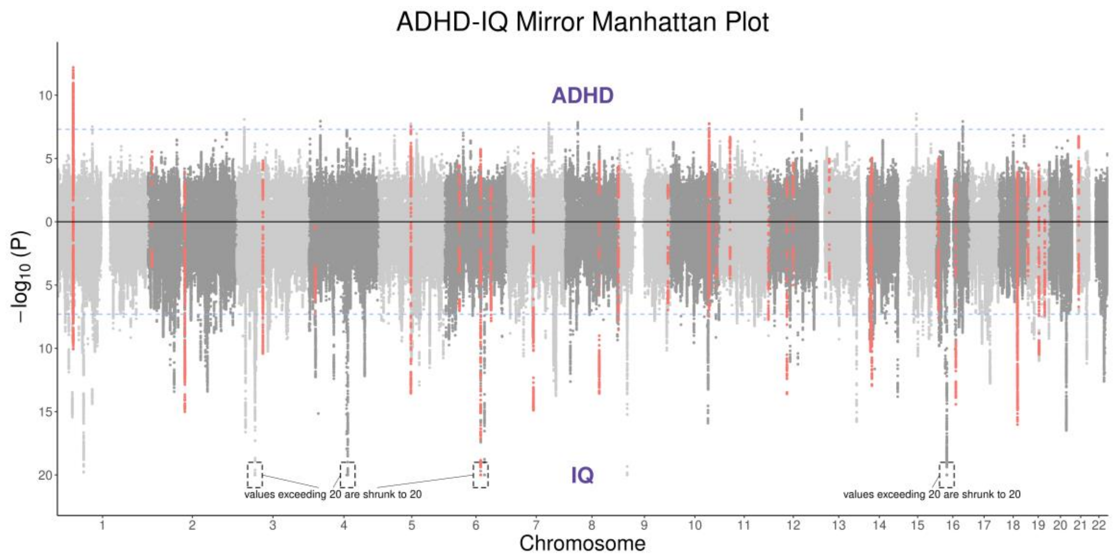

Supplementary Figure 27. Mirror Manhattan plot for ADHD-IQ. Red dots represent SNPs located in the LOGODetect detected regions. For IQ, four loci on chromosome 3, 4, 6, and 16 have $-\log_{10} P$ value exceeding 20, those values are shrunk to 20 for conciseness.

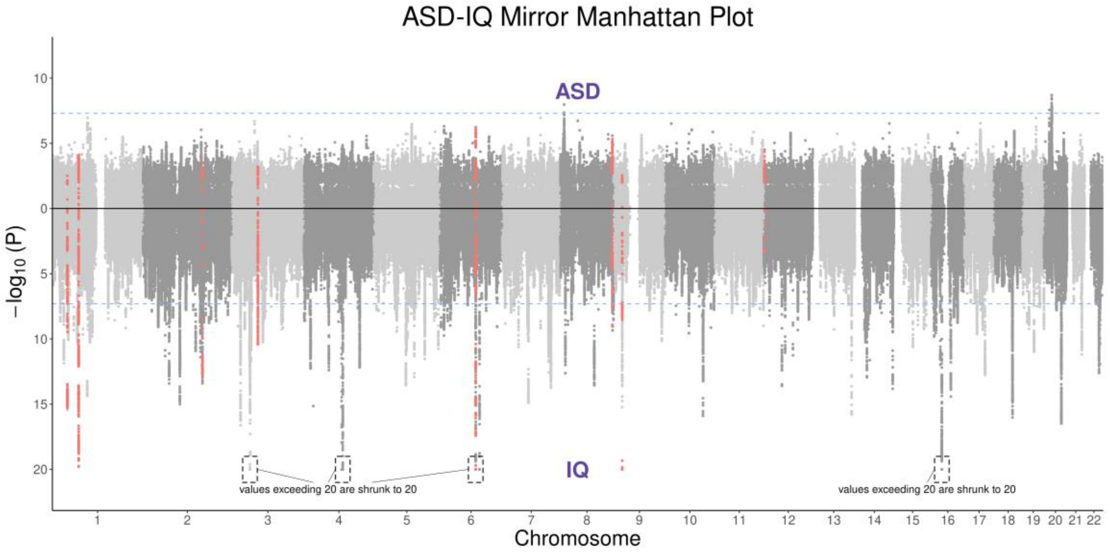

Supplementary Figure 28. Mirror Manhattan plot for ASD-IQ. Red dots represent SNPs located in the LOGODetect detected regions. For IQ, four loci on chromosome 3, 4, 6, and 16 have $-\log_{10} P$ value exceeding 20, those values are shrunk to 20 for conciseness.

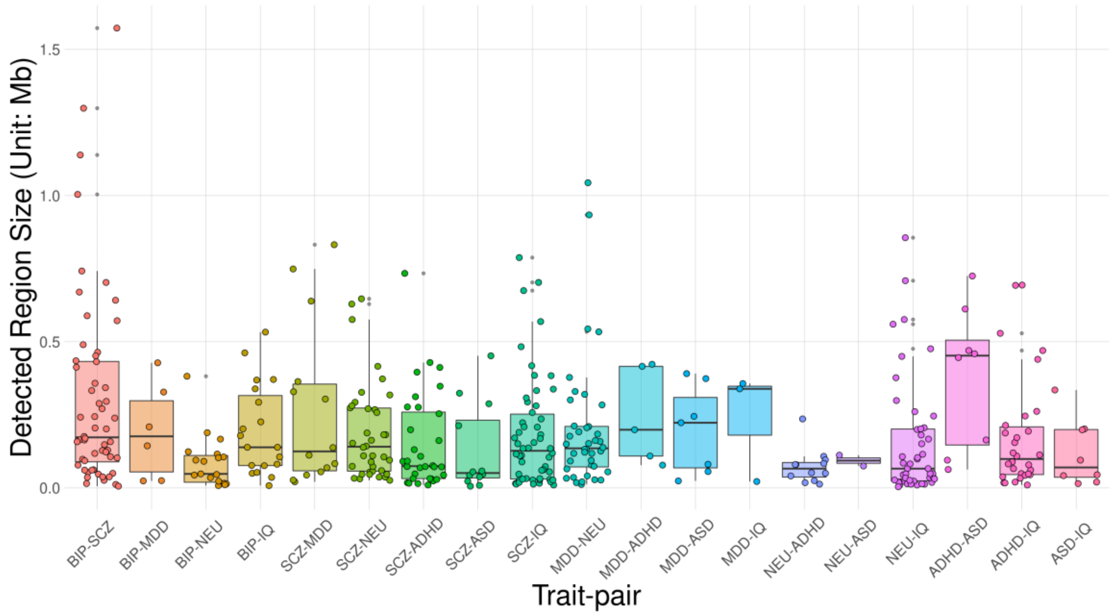

Supplementary Figure 29. Box plot of detected region size identified by LOGODetect in different trait-pairs. Only 19 trait-pairs with detected significant regions are shown.

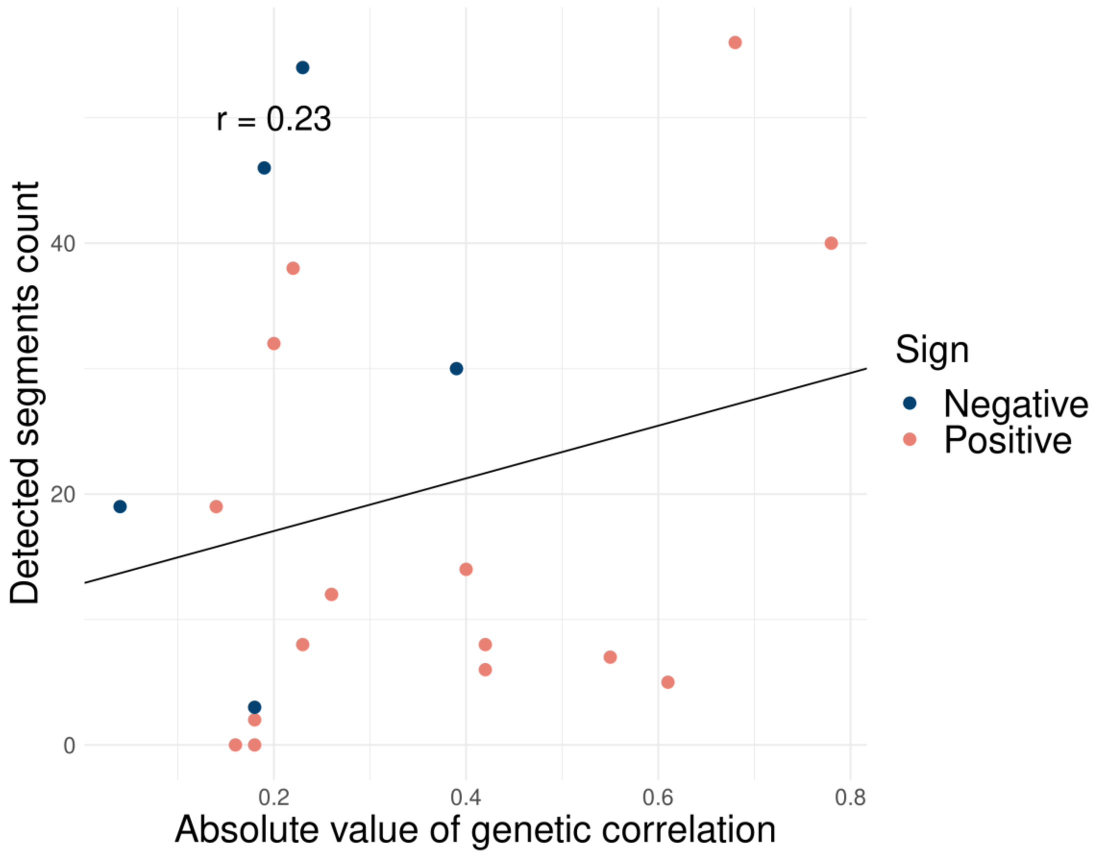

Supplementary Figure 30. Number of segments identified by LOGODetect and the absolute value of genetic correlation estimated by cross-trait LDSC are concordant (correlation r=0.23). The larger the absolute genetic correlation, the more segments detected by LOGODetect.

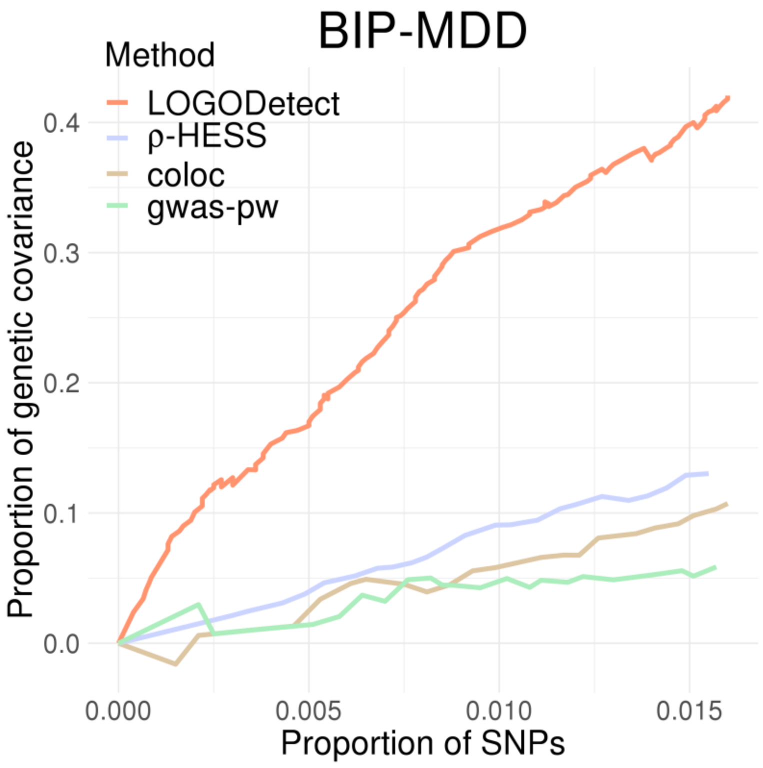

Supplementary Figure 31. Genetic covariance explained by the same number of SNPs in regions identified by four methods. Here two traits are BIP and MDD.

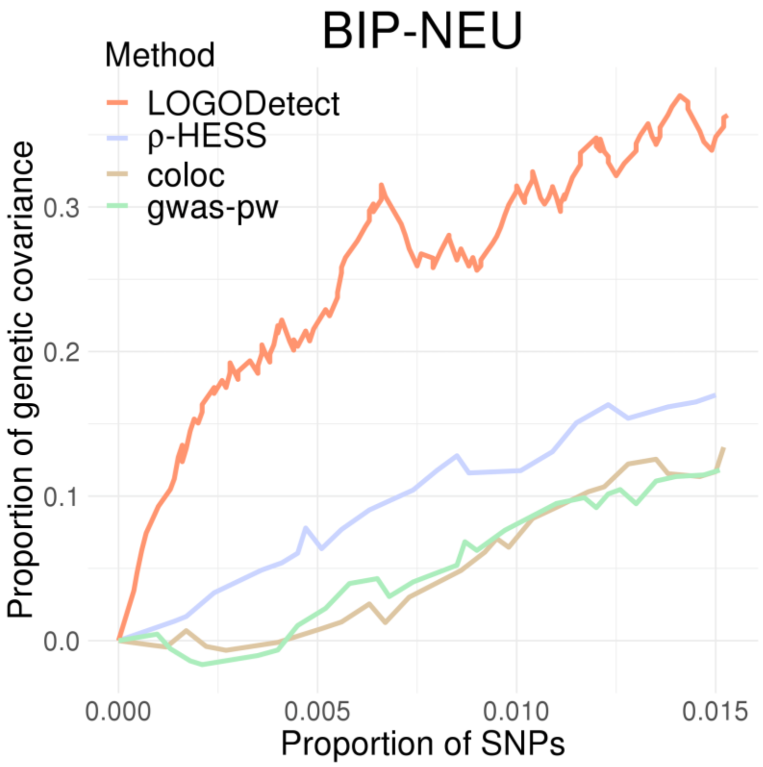

Supplementary Figure 32. Genetic covariance explained by the same number of SNPs in regions identified by four methods. Here two traits are BIP and NEU.

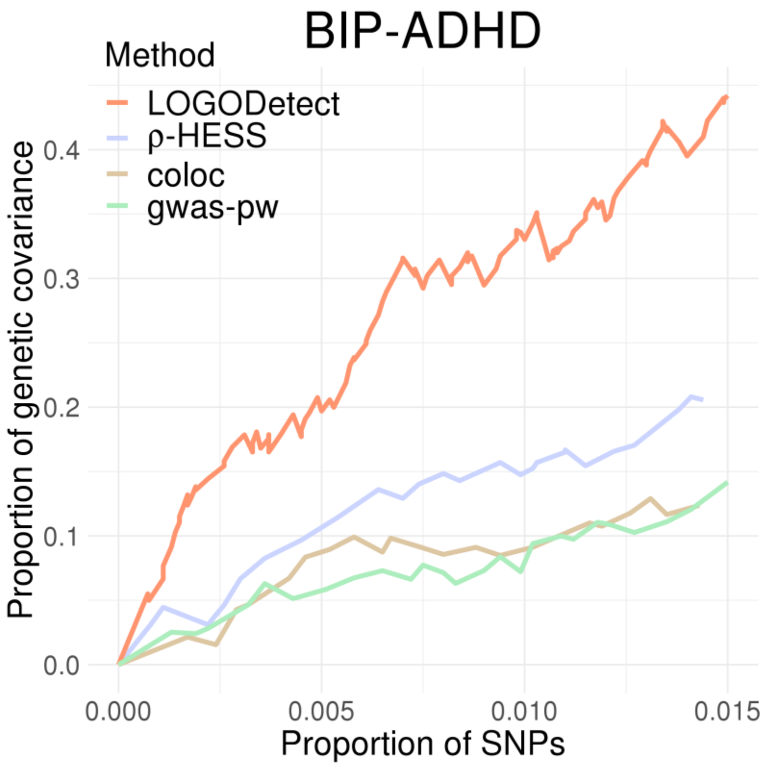

Supplementary Figure 33. Genetic covariance explained by the same number of SNPs in regions identified by four methods. Here two traits are BIP and ADHD.

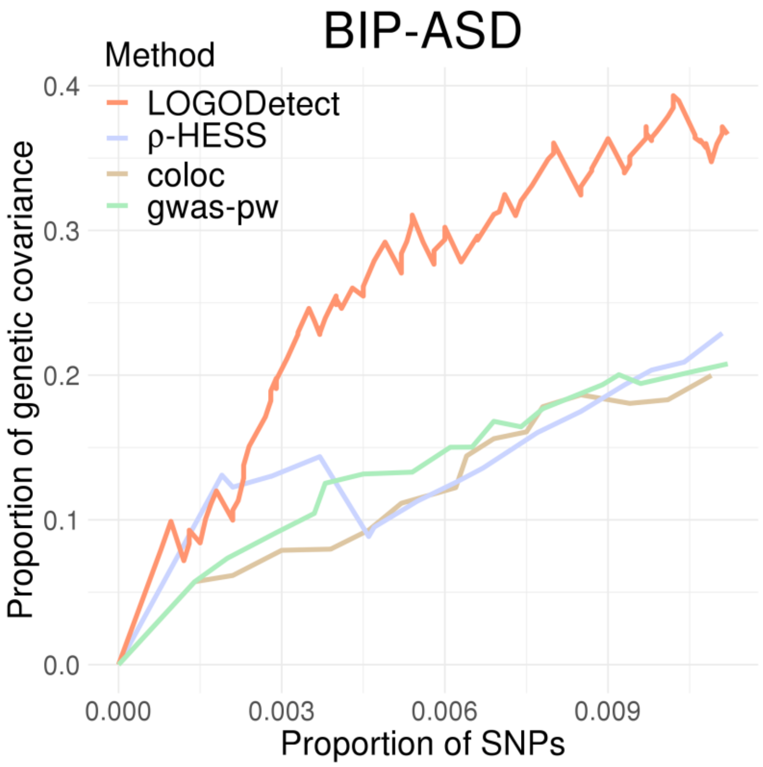

Supplementary Figure 34. Genetic covariance explained by the same number of SNPs in regions identified by four methods. Here two traits are BIP and ASD.

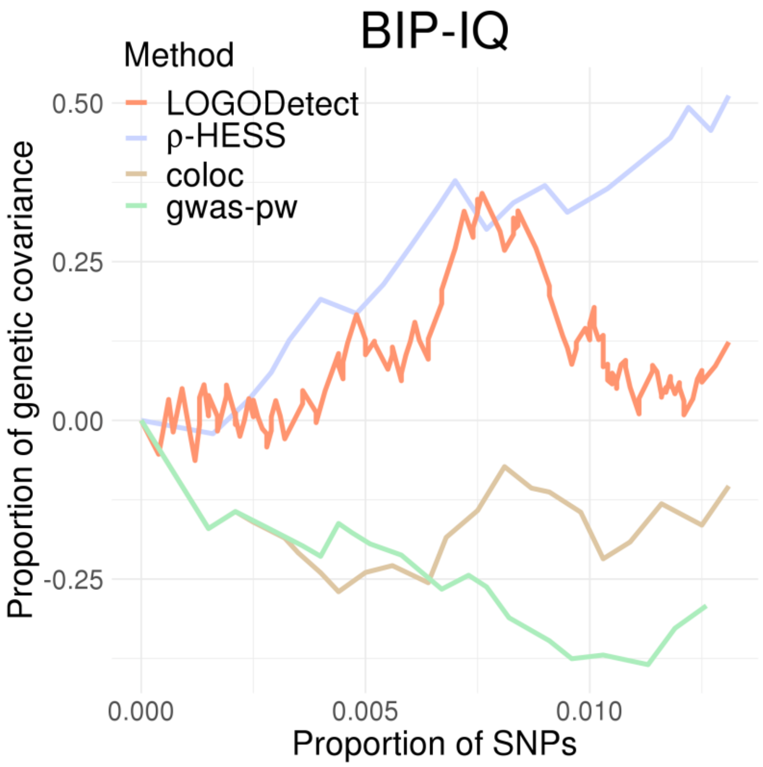

Supplementary Figure 35. Genetic covariance explained by the same number of SNPs in regions identified by four methods. Here two traits are BIP and IQ. Genetic correlation is not significant between BIP and IQ (r_g = -0.04, p=0.12). Part of top regions identified by LOGODetect have positive local genetic correlation while others have negative local genetic correlation. Aggregating these regions together, their contribution to genetic covariance will cancel out and lead to non-significant genetic covariance and genetic correlation.

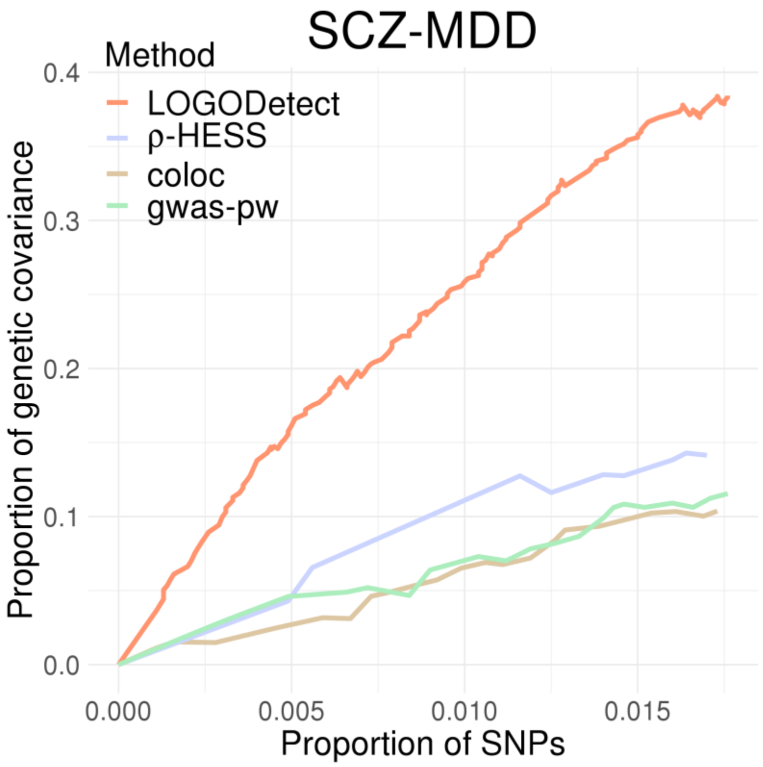

Supplementary Figure 36. Genetic covariance explained by the same number of SNPs in regions identified by four methods. Here two traits are SCZ and MDD.

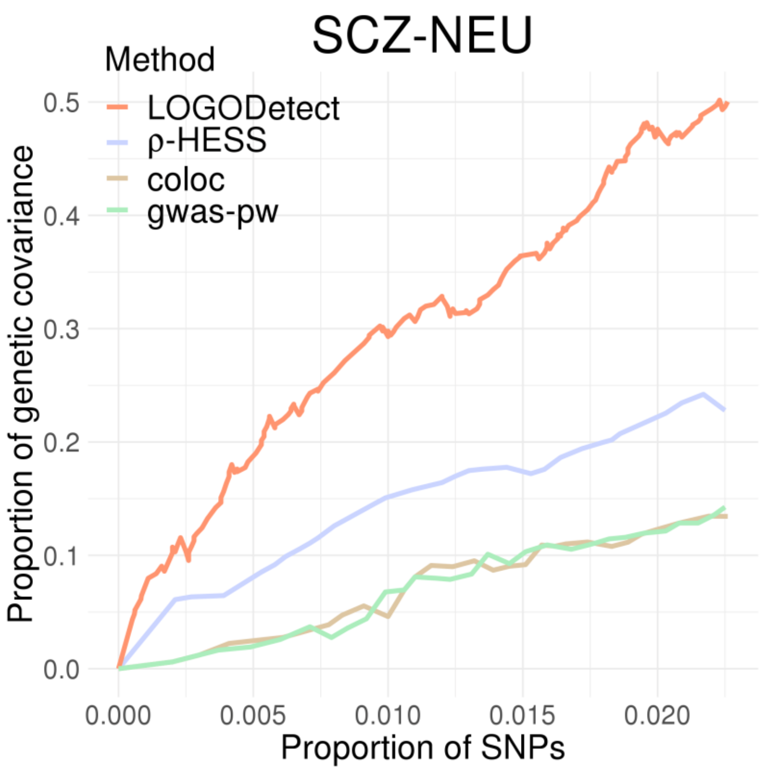

Supplementary Figure 37. Genetic covariance explained by the same number of SNPs in regions identified by four methods. Here two traits are SCZ and NEU.

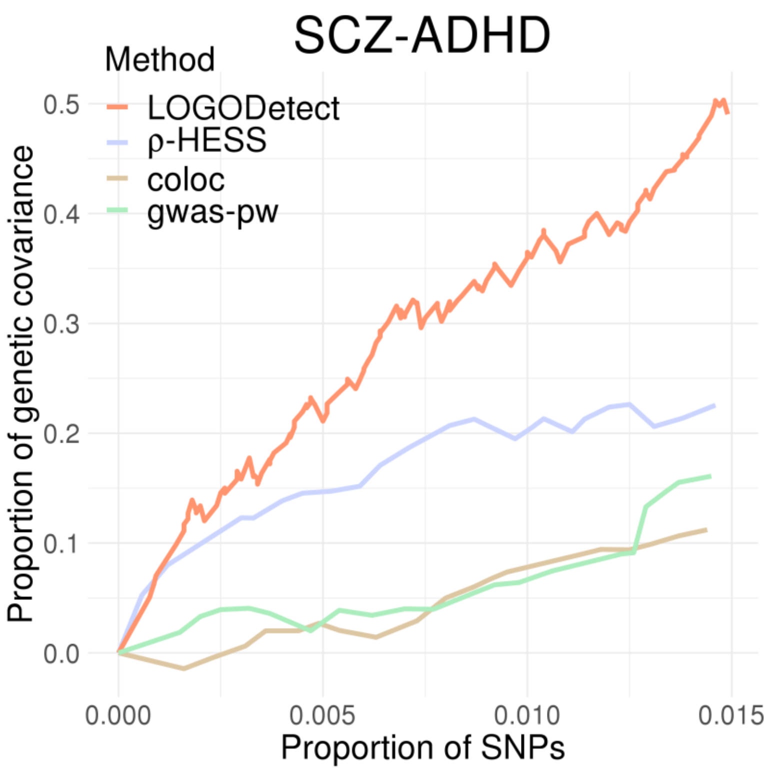

Supplementary Figure 38. Genetic covariance explained by the same number of SNPs in regions identified by four methods. Here two traits are SCZ and ADHD.

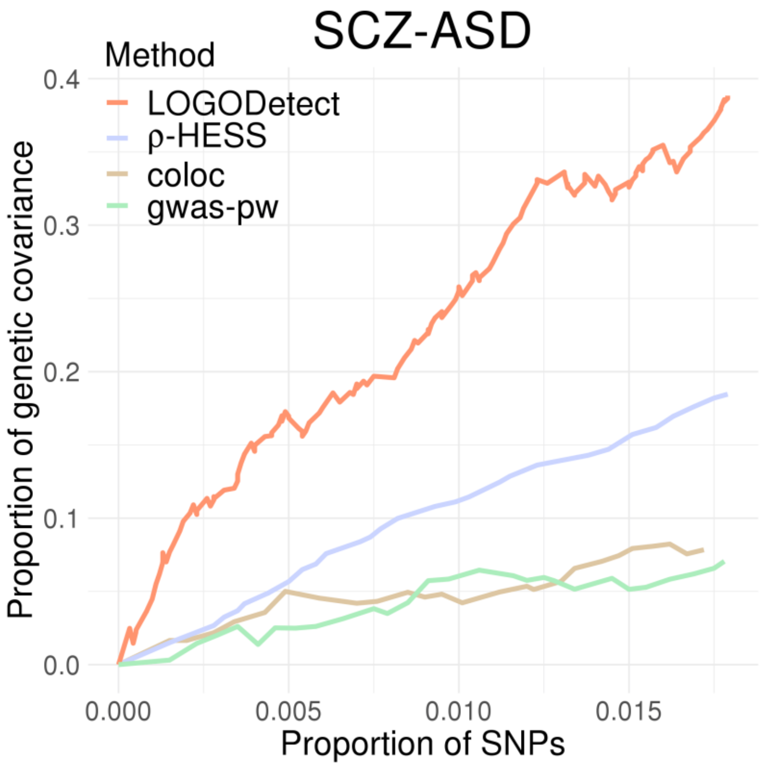

Supplementary Figure 39. Genetic covariance explained by the same number of SNPs in regions identified by four methods. Here two traits are SCZ and ASD.

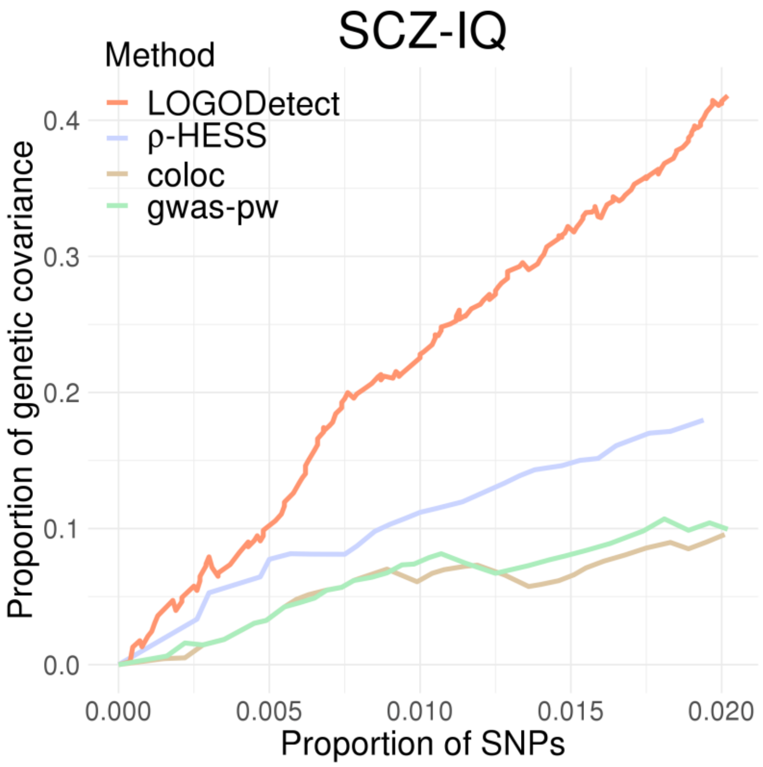

Supplementary Figure 40. Genetic covariance explained by the same number of SNPs in regions identified by four methods. Here two traits are SCZ and IQ.

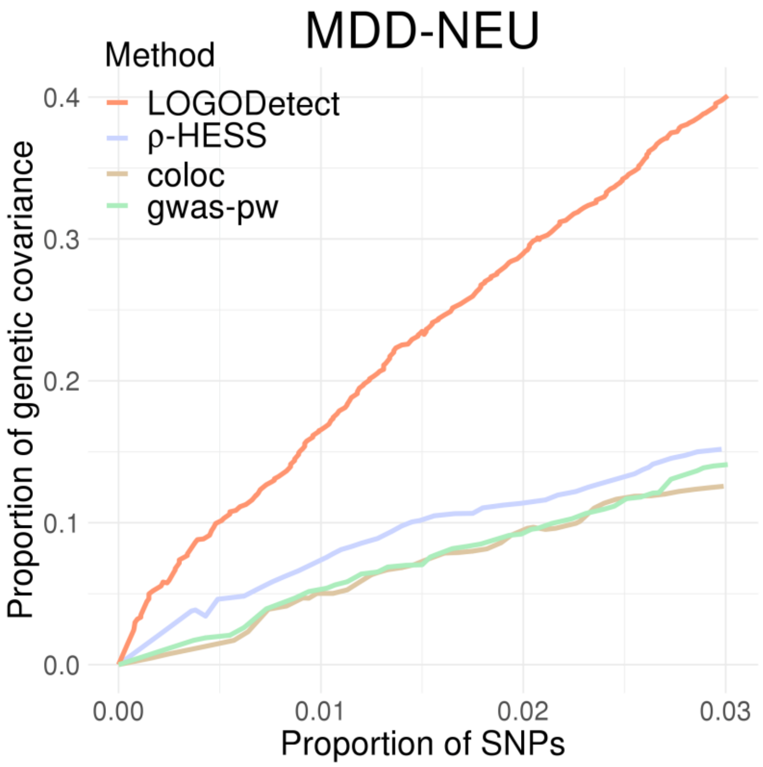

Supplementary Figure 41. Genetic covariance explained by the same number of SNPs in regions identified by four methods. Here two traits are MDD and NEU.

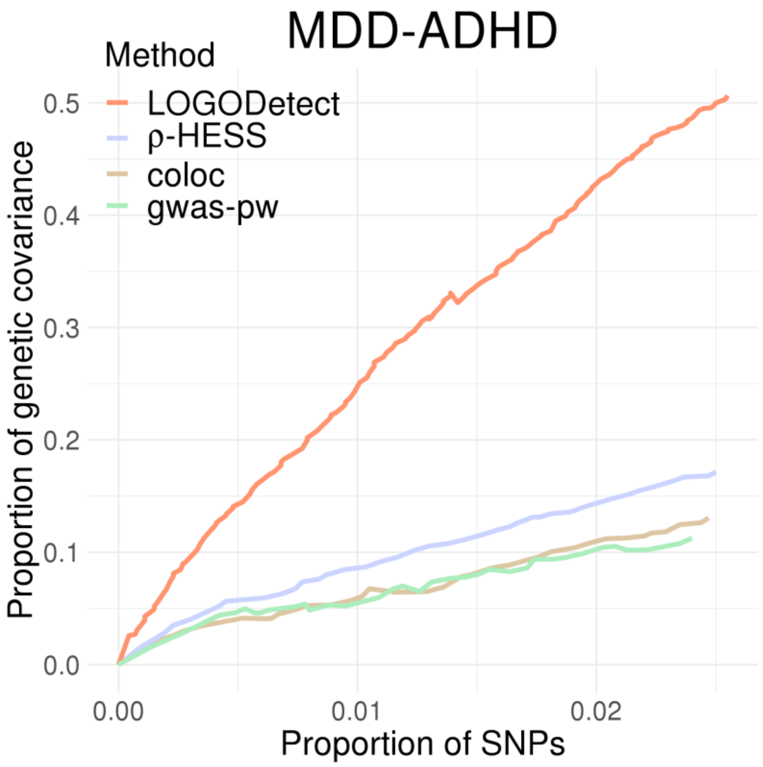

Supplementary Figure 42. Genetic covariance explained by the same number of SNPs in regions identified by four methods. Here two traits are MDD and ADHD.

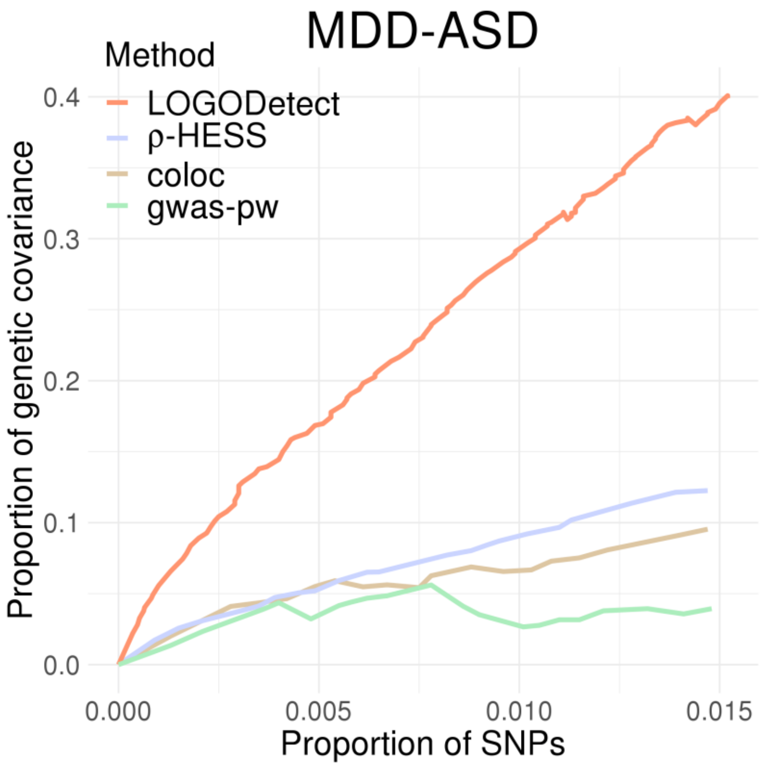

Supplementary Figure 43. Genetic covariance explained by the same number of SNPs in regions identified by four methods. Here two traits are MDD and ASD.

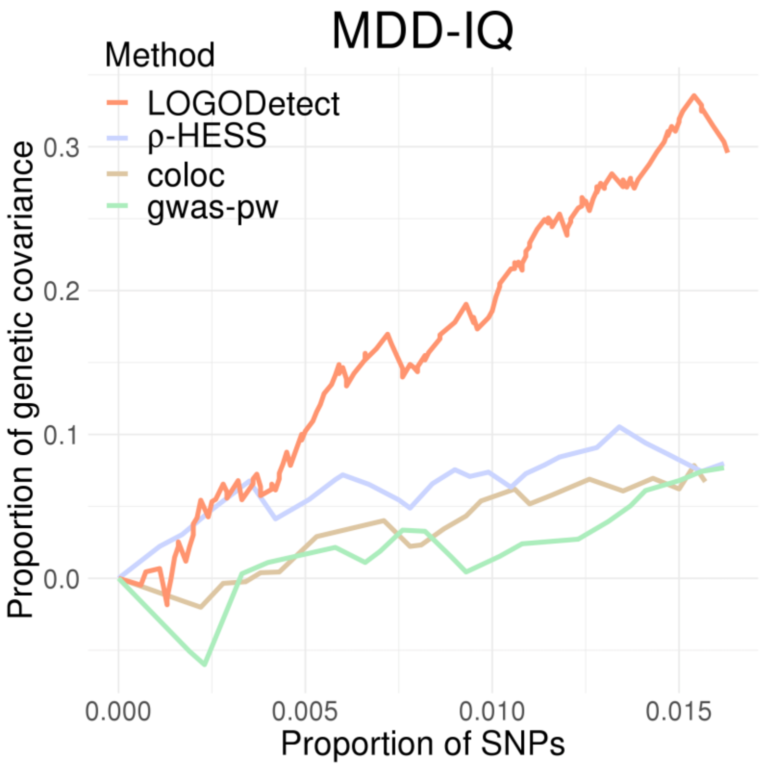

Supplementary Figure 44. Genetic covariance explained by the same number of SNPs in regions identified by four methods. Here two traits are MDD and IQ.

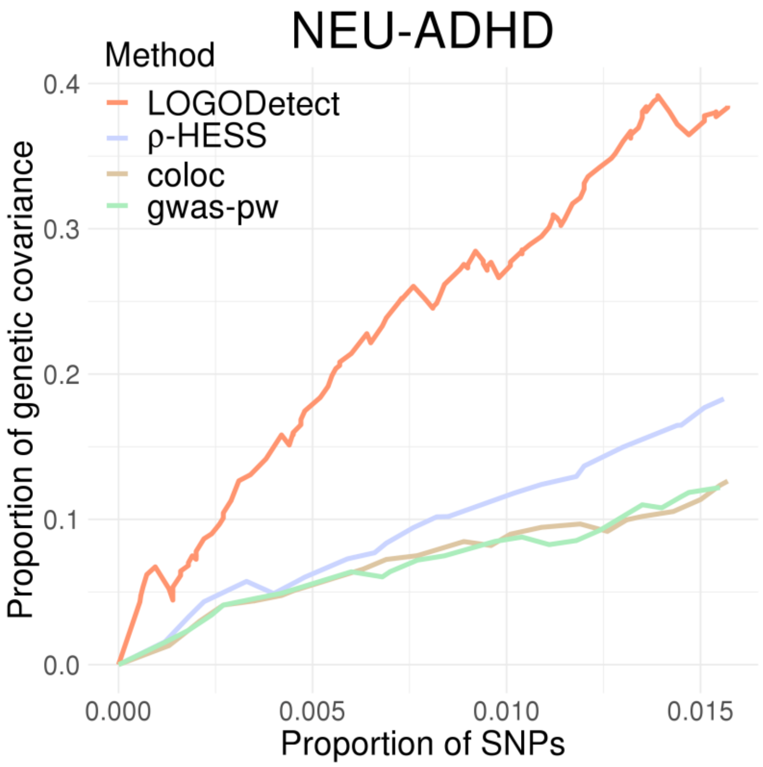

Supplementary Figure 45. Genetic covariance explained by the same number of SNPs in regions identified by four methods. Here two traits are NEU and ADHD.

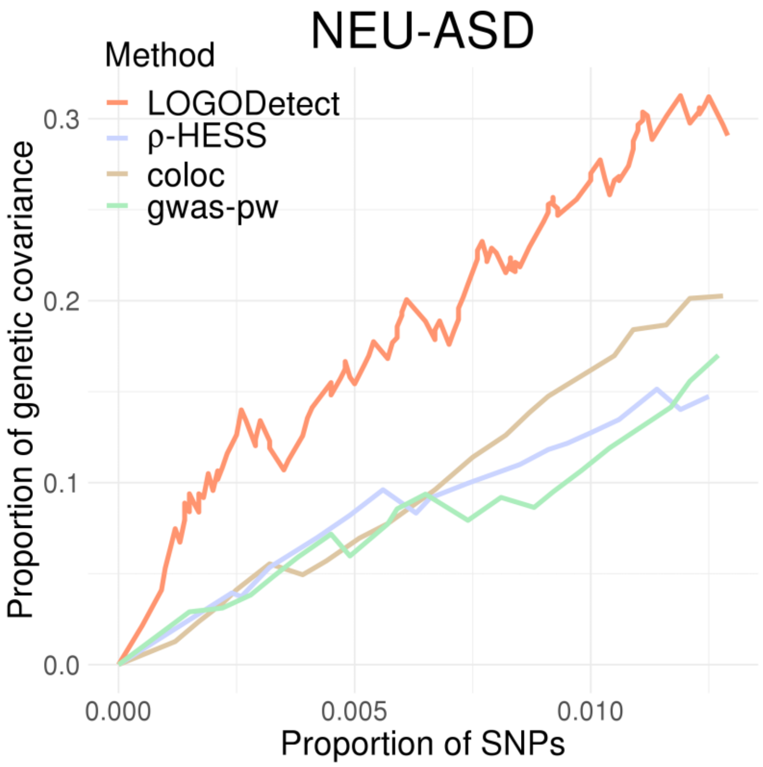

Supplementary Figure 46. Genetic covariance explained by the same number of SNPs in regions identified by four methods. Here two traits are NEU and ASD.

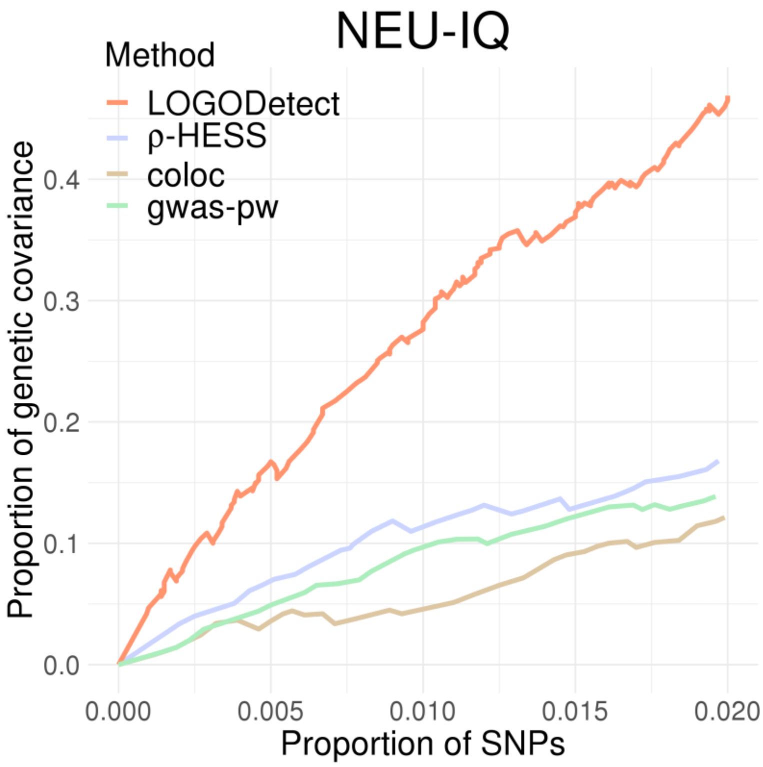

Supplementary Figure 47. Genetic covariance explained by the same number of SNPs in regions identified by four methods. Here two traits are NEU and IQ.

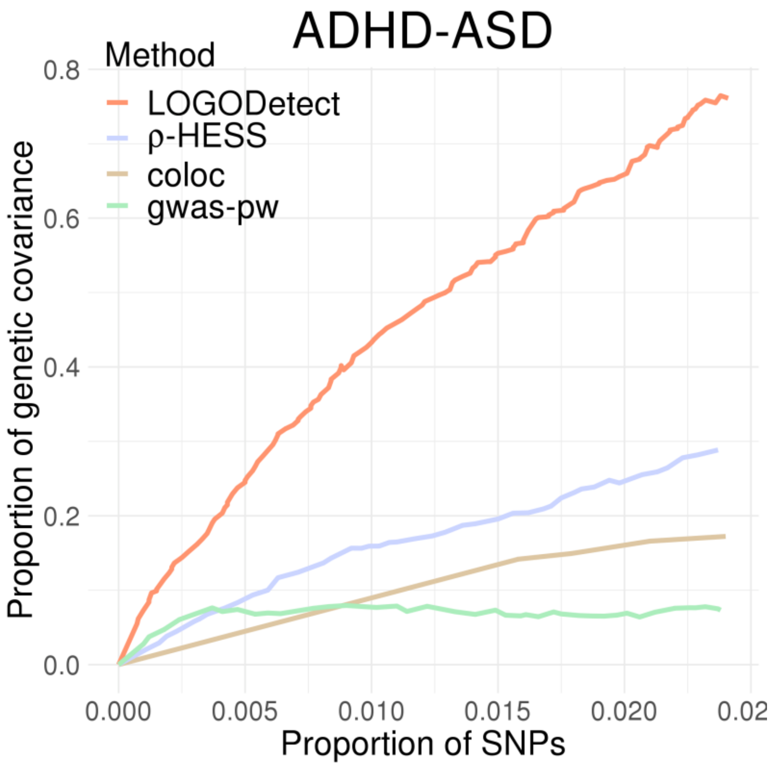

Supplementary Figure 48. Genetic covariance explained by the same number of SNPs in regions identified by four methods. Here two traits are ADHD and ASD.

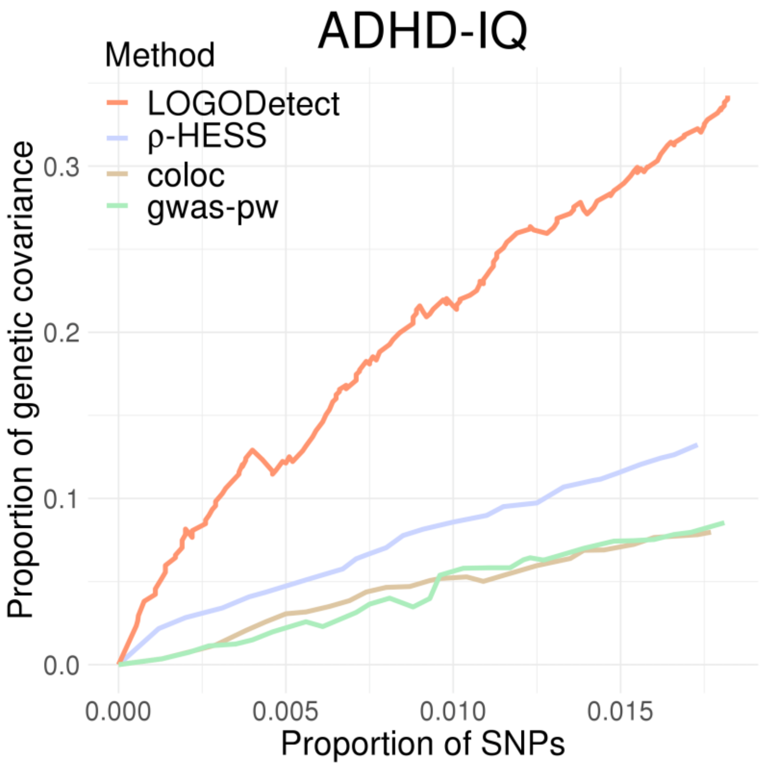

Supplementary Figure 49. Genetic covariance explained by the same number of SNPs in regions identified by four methods. Here two traits are ADHD and IQ.

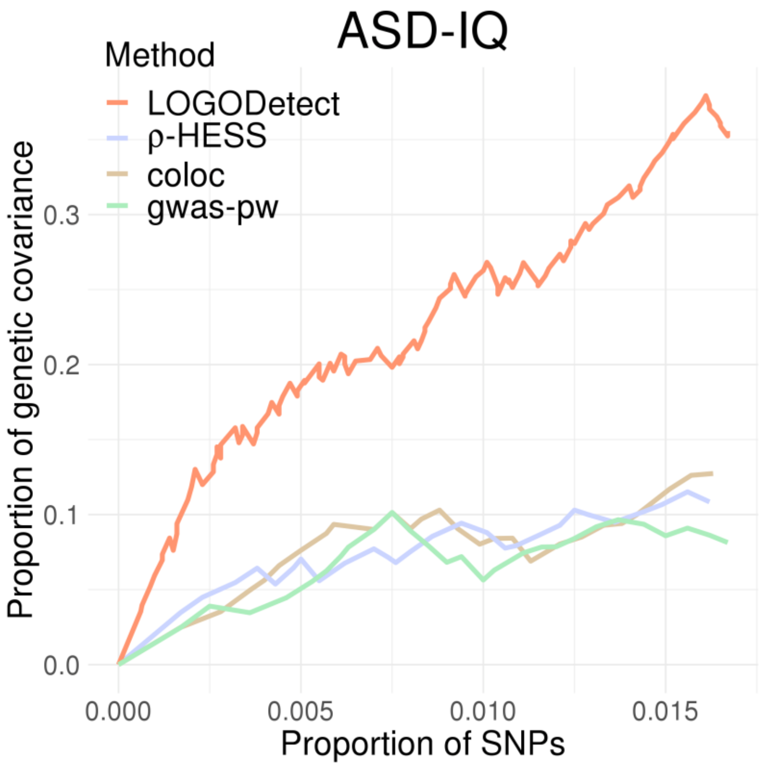

Supplementary Figure 50. Genetic covariance explained by the same number of SNPs in regions identified by four methods. Here two traits are ASD and IQ.

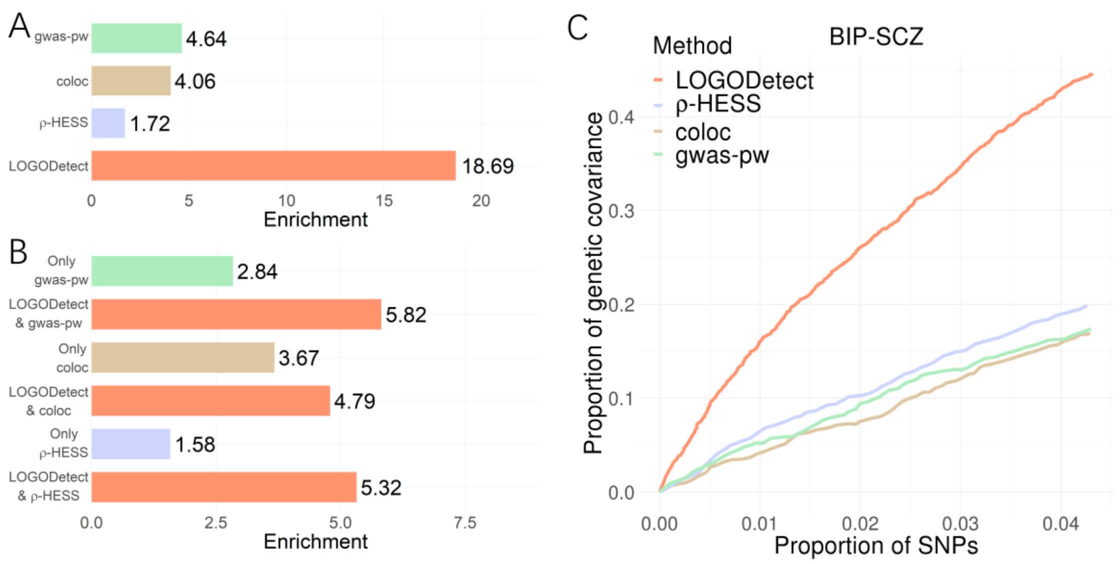

Supplementary Figure 51. LOGODetect identifies precise genomic regions harboring local genetic correlations. Genetic covariance and its corresponding enrichment were calculated using stratified LDSC. (A) Genetic covariance fold enrichment (i.e. the ratio between the proportion of total genetic covariance and the proportion of the total SNP counts) in regions identified by LOGODetect, ρ-HESS, coloc, and gwas-pw respectively. (B) Genetic covariance fold enrichment in regions identified by ρ-HESS, coloc, and gwas-pw that also overlapped with LOGODetect findings, and regions identified by ρ-HESS, coloc, and gwas-pw alone. (C) Genetic covariance explained by the same number of SNPs in regions identified by four methods. Here we used estimated overlapping sample sizes to de-bias ρ-HESS results.

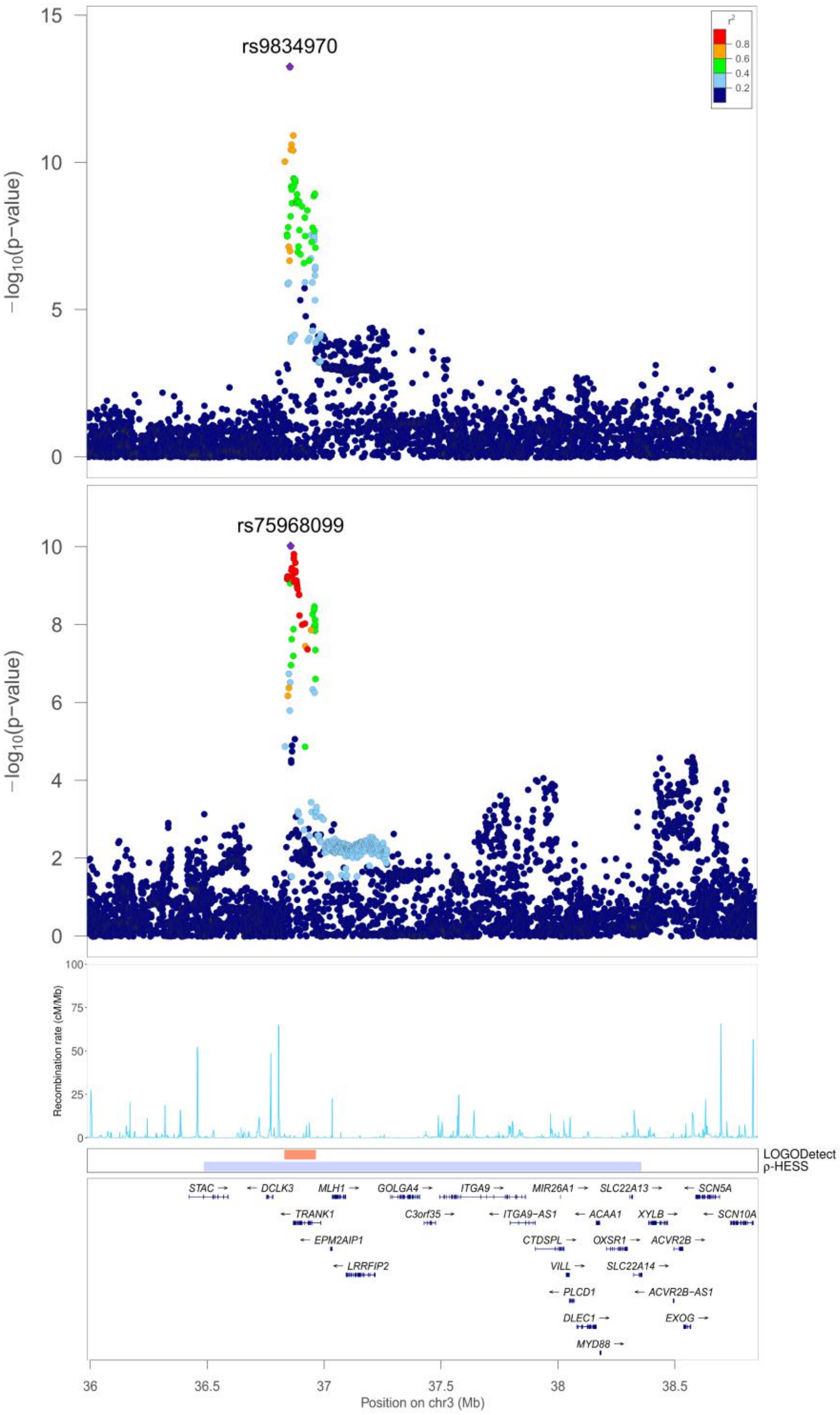

Supplementary Figure 52. LOGODetect identify signal regions more precisely than other methods. Two locuszoom plots are shown, the above one is BIP, the other one is SCZ. The orange band represent the region identified by LOGODetect, the light purple band represent the pre-specified region (defined by independent LD blocks) used in ρ-HESS and colocalization methods.

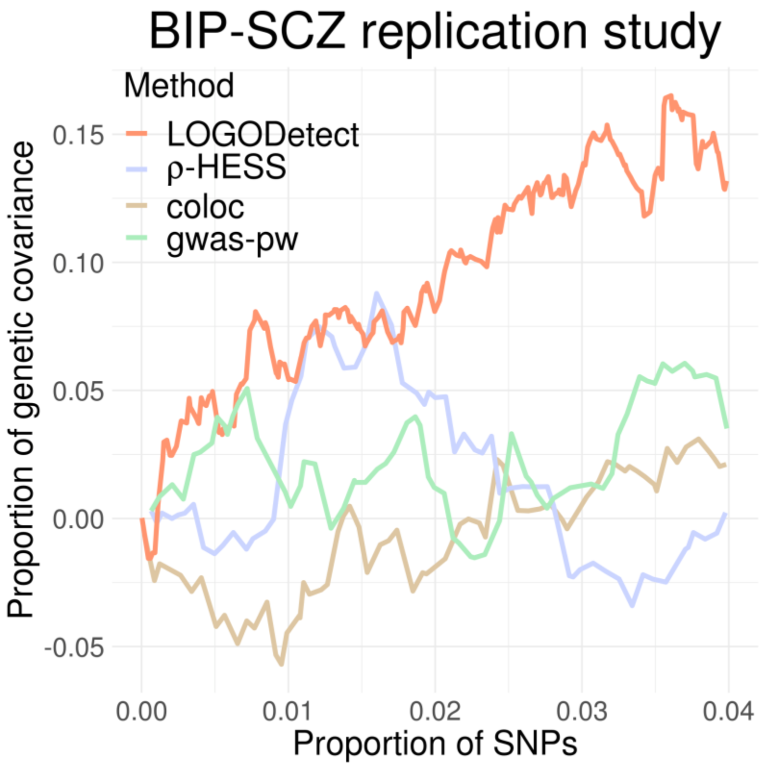

Supplementary Figure 53. Genetic covariance enrichment curve of the identified regions for the two methods at varying threshold. Here two traits are BIP and SCZ in replication cohorts (UKBB).

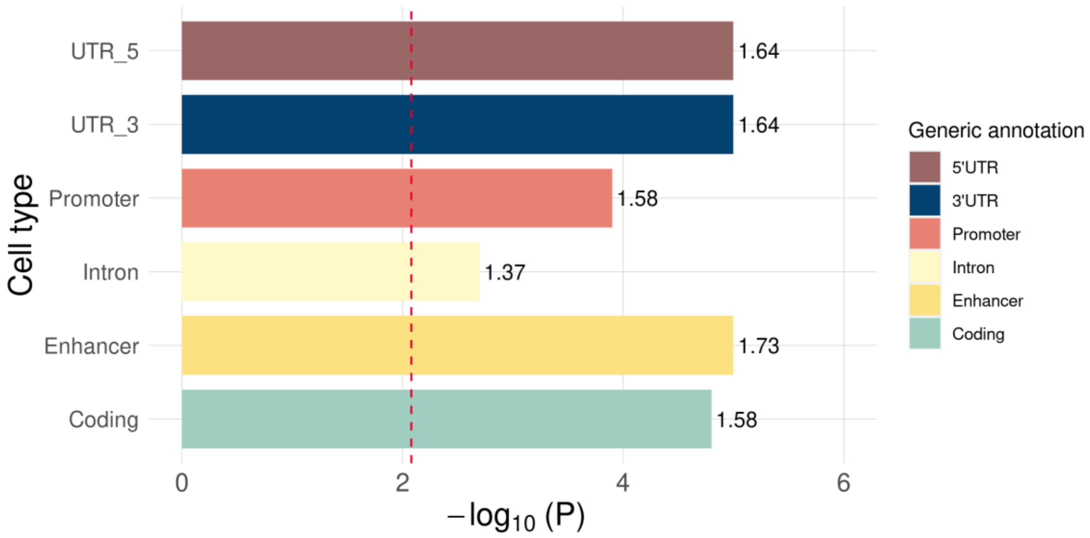

Supplementary Figure 54. Enrichment in the predicted functional regions in brain tissues after conditioning on the annotation overlap with six generic annotations.

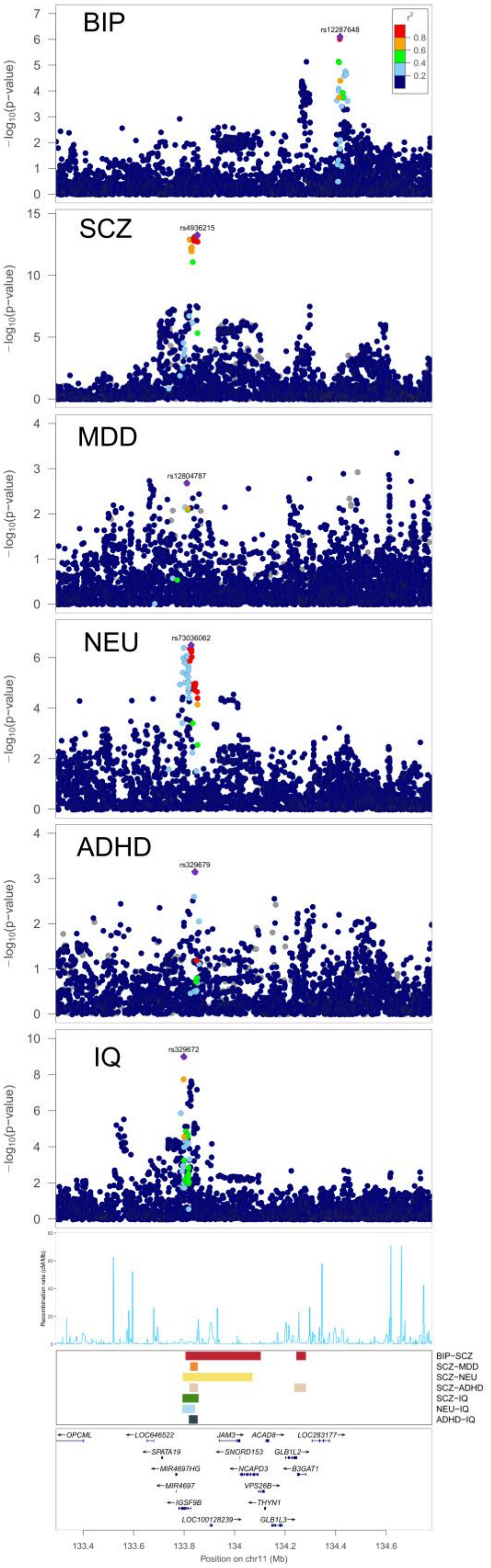

Supplementary Figure 55. Putative target genes for the hub region in chr11 shared by seven neuropsychiatric trait pairs. Locuszoom plot, recombination rate, and the gene names are provided. The colored band denote the location of the significant region and which trait pair is detected in.

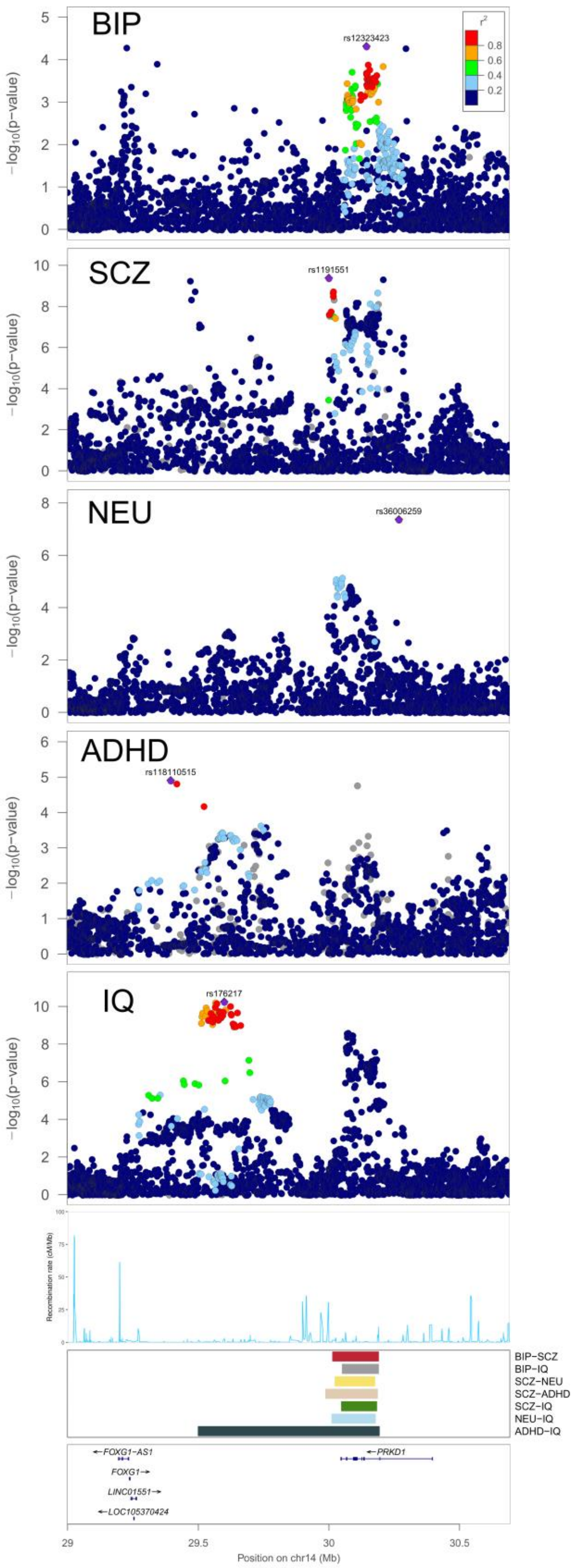

Supplementary Figure 56. Putative target genes for the hub region in chr14 shared by seven neuropsychiatric trait pairs. Locuszoom plot, recombination rate, and the gene names are provided. The colored band denote the location of the significant region and which trait pair is detected in.

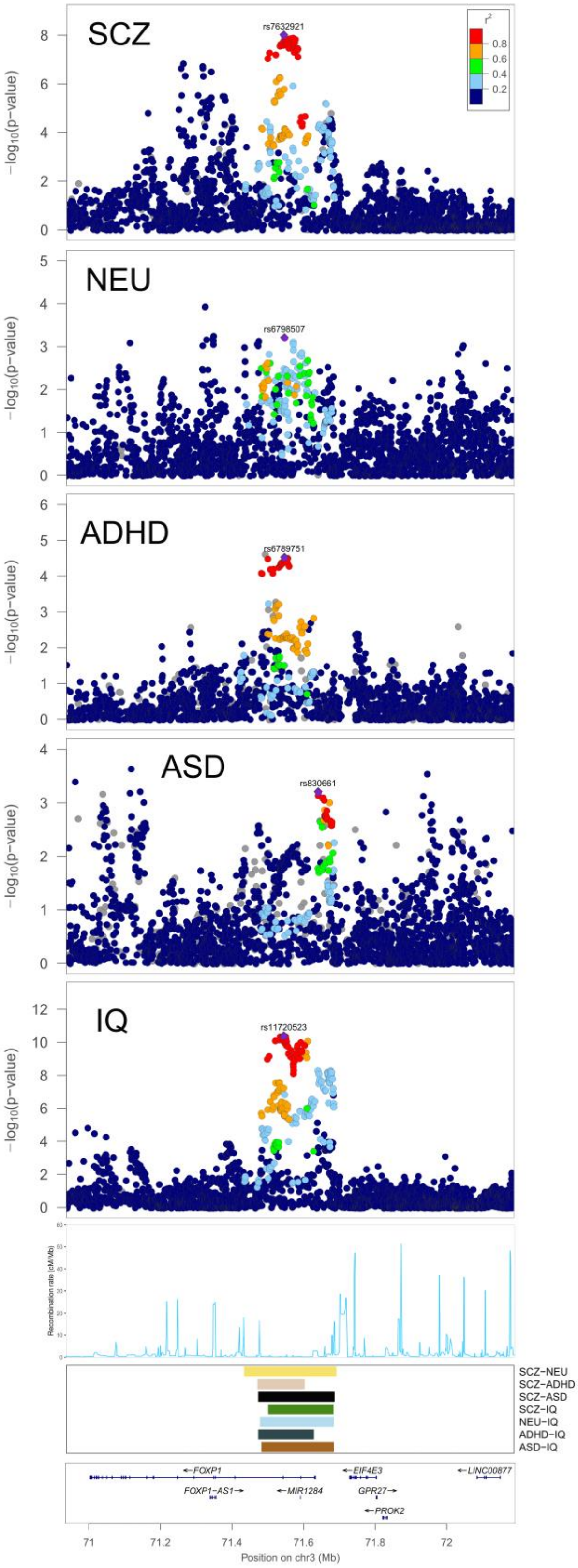

Supplementary Figure 57. Putative target genes for the hub region in chr3 shared by seven neuropsychiatric trait pairs. Locuszoom plot, recombination rate, and the gene names are provided. The colored band denote the location of the significant region and which trait pair is detected in.

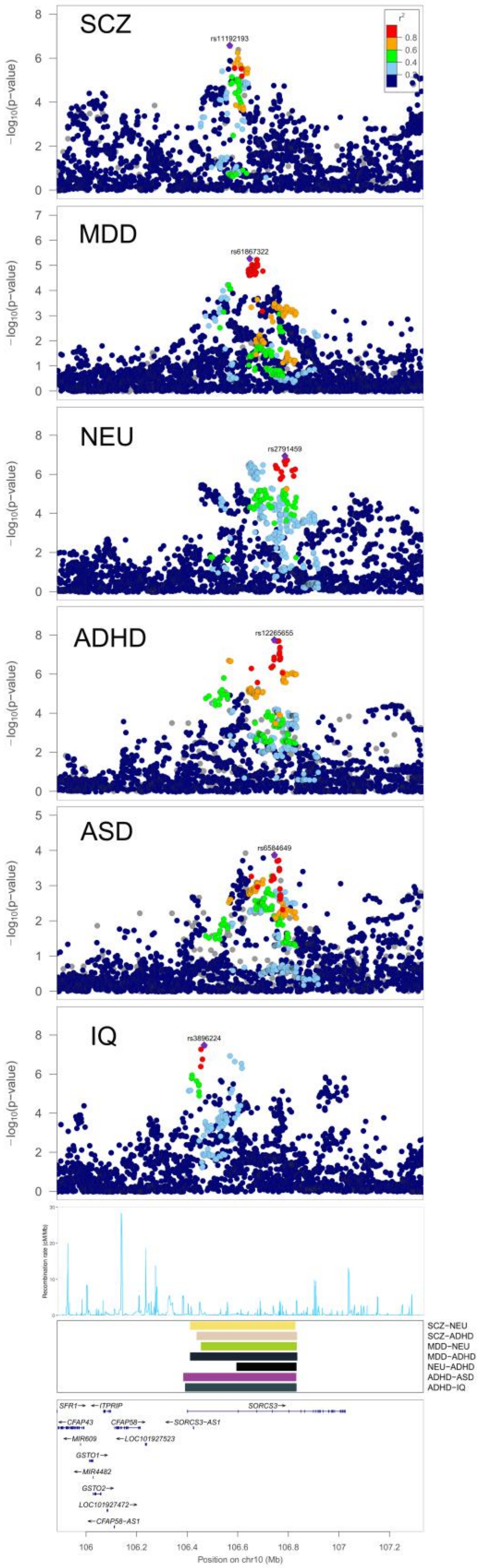

Supplementary Figure 58. Putative target genes for the hub region in chr10 shared by seven neuropsychiatric trait pairs. Locuszoom plot, recombination rate, and the gene names are provided. The colored band denote the location of the significant region and which trait pair is detected in.

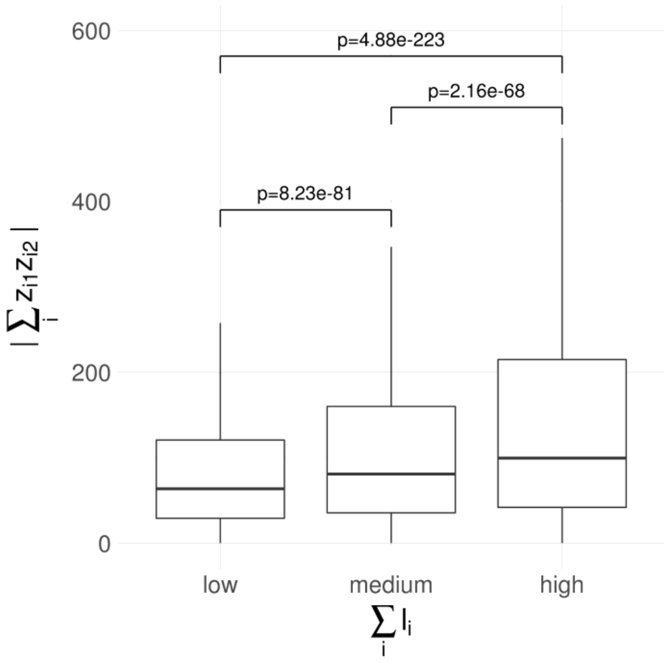

Supplementary Figure 59. $|\sum_{i \in R} z_{1i} z_{2i}|$ **is larger in regions with strong LD for BIP and SCZ.** Genome is partitioned into 30,957 blocks, each containing 200 SNPs. Blocks are classified into three categories according to the LD strength (sum of LD scores), each category has 10,319 independent blocks. For each category pair, one-sided two-sample t-test is performed and the corresponding p value is provided. Here, the bottom and upper end of the whisker denote the minima and maxima respectively, the bottom, middle, and upper line of the box denote the first quartile Q_1, median Q_2, and the third quartile Q_3 respectively, and outliers (data points that fall outside $[Q_1 - 1.5 * IQR,\ Q_3 + 1.5 * IQR]$, where $IQR = Q_3 - Q_1$) are omitted for visualization purposes.

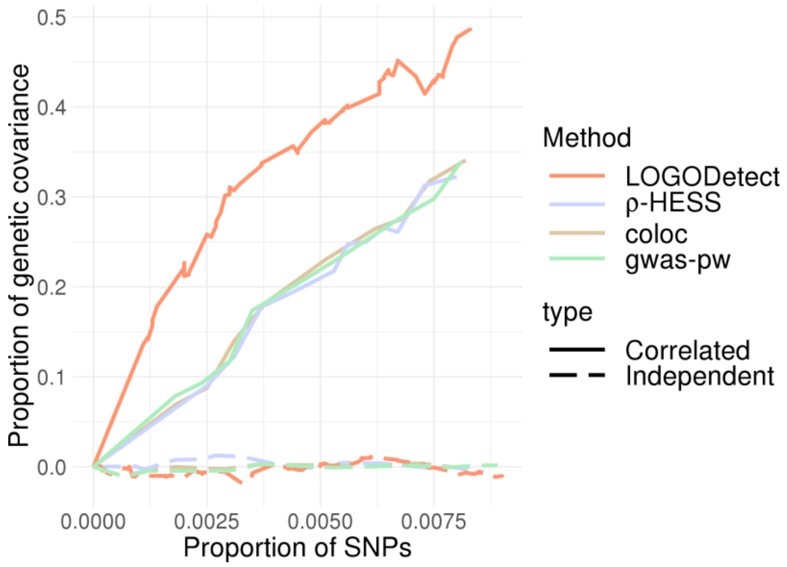

Supplementary Figure 60. Genetic covariance enrichment curve in simulations. Two genetic correlated traits are simulated and the corresponding top regions are identified using four methods. Regions identified in the two correlated traits have significant genetic covariance enrichment with respect to the two correlated traits, as depicted in solid line. We simulate another two independent traits and obtain the corresponding top regions using four methods. Regions identified in the two independent traits have no genetic covariance enrichment with respect to the two correlated traits, as depicted in dashed line.

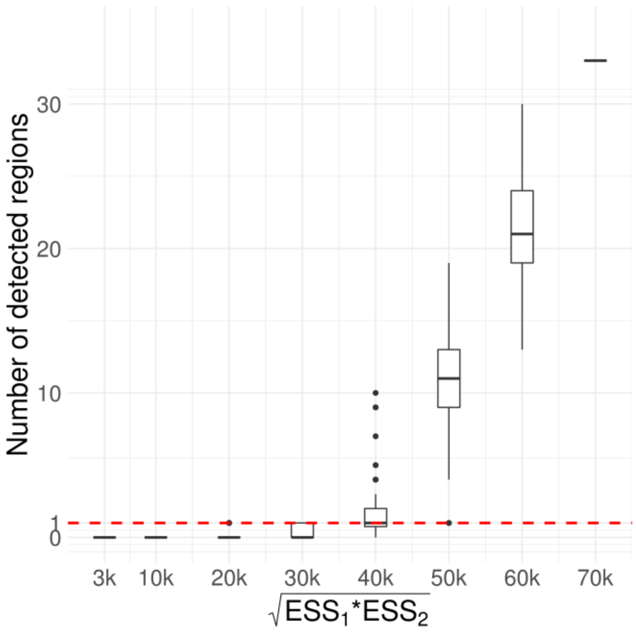

Supplementary Figure 61. Statistical power decreases as effective sample size decreases. Here the X-axis denotes the squared root of the effective sample size of the simulated BIP cohort times that of the simulated SCZ cohort. The Y-axis denotes the number of regions detected by LOGODetect. The box on the right corresponds to effective sample size in the BIP-SCZ discovery cohorts ($\sqrt{ESS_1 * ESS_2} = 70{,}214$), the box on the left corresponds to effective sample size in the BIP-SCZ UKBB replication cohorts ($\sqrt{ESS_1 * ESS_2} = 3{,}111$). Each box represents 100 independent simulations for each effective sample size. Here, the bottom and upper end of the whisker denote the minima and maxima respectively, the bottom, middle, and upper line of the box denote the first quartile Q_1, median Q_2, and the third quartile Q_3 respectively, and the dots beyond the whiskers denote outliers (data points that fall outside $[Q_1 - 1.5 * IQR,\ Q_3 + 1.5 * IQR]$, where $IQR = Q_3 - Q_1$).

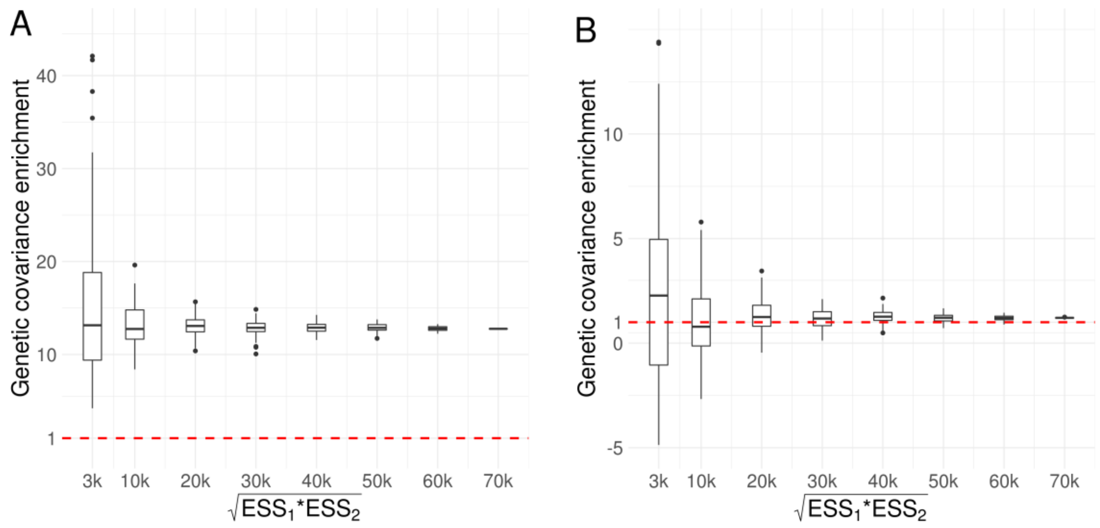

Supplementary Figure 62. Enrichment of aggregated genetic covariance with varying effective sample size. (A) Regions identified by LOGODetect in discovery cohort show substantial genetic covariance enrichment in UKBB replication cohort. (B) Randomly selected regions with same sizes as (A) show no genetic covariance enrichment in UKBB replication cohort. Genetic covariance of randomly selected regions may have opposite sign compared to global genetic covariance, therefore the corresponding genetic covariance enrichment may be negative. Here the X-axis denotes the squared root of the effective sample size of the simulated BIP cohort times that of the simulated SCZ cohort. For each panel, the box on the right corresponds to effective sample size in the BIP-SCZ discovery cohorts ($\sqrt{ESS_1 * ESS_2} = 70{,}214$), the box on the left corresponds to effective sample size in the BIP-SCZ UKBB replication cohorts ($\sqrt{ESS_1 * ESS_2} = 3{,}111$). Each box represents 100 independent simulations for each effective sample size. Here, the bottom and upper end of the whisker denote the minima and maxima respectively, the bottom, middle, and upper line of the box denote the first quartile Q_1, median Q_2, and the third quartile Q_3 respectively, and the dots beyond the whiskers denote outliers (data points that fall outside $[Q_1 - 1.5 * IQR,\ Q_3 + 1.5 * IQR]$, where $IQR = Q_3 - Q_1$).

Supplementary Tables

Supplementary Table 1. Type I errors under an infinitesimal model

	Type I error rate at significance level α=0.05			Empirical type I error rate at posterior probability cutoff 0.95	
Trait heritability	LOGODetect	ρ-HESS		coloc	gwas-pw
0.05	0.04	0		0	0.46
0.04	0.02	0		0	0.35
0.03	0.06	0		0	0.16
0.02	0.04	0		0	0.07
0.01	0.02	0		0	0

Type I error rates and empirical type I error rates denote the proportion of simulations that significant segments harboring local genetic correlation are identified under the null. Here we assume an infinitesimal model where per-SNP heritability for all the SNPs are the same. Two cohorts do not have sample overlaps. Simulations under different parameters scenarios are repeated for 100 times.

Supplementary Table 2. Type I errors under a heritability enrichment model

	Type I error rate at significance level α=0.05			Empirical type I error rate at posterior probability cutoff 0.95	
Trait heritability	LOGODetect	ρ-HESS		coloc	gwas-pw
0.05	0.05	0		0.01	0.45
0.04	0.02	0		0	0.23
0.03	0.03	0		0	0.18
0.02	0.03	0		0	0.05
0.01	0	0		0	0.01

Type I error rates and empirical type I error rates denote the proportion of simulations that significant segments harboring local genetic correlation are identified under the null. Here we assume a heritability enrichment model that 30% trait heritability assigned to 5000 randomly selected SNPs and 70% trait heritability assigned to other SNPs. Two cohorts do not have sample overlaps. Simulations under different parameters scenarios are repeated for 100 times.

Supplementary Table 3. Type I errors under a LDAK model

	Type I error rate at significance level α=0.05			Empirical type I error rate at posterior probability cutoff 0.95	
Trait heritability	LOGODetect	ρ-HESS		coloc	gwas-pw
0.05	0.03	0		0	0.09
0.04	0.01	0		0	0.06
0.03	0.02	0		0	0.03
0.02	0.01	0		0	0.02
0.01	0	0		0	0

Type I error rates and empirical type I error rates denote the proportion of simulations that significant segments harboring local genetic correlation are identified under the null. Here we assume a LDAK genetic architecture, where the j-th SNP heritability is proportional to $[f_j(1-f_j)]^{0.75} * w_j$, f_j and w_j denote minor allele frequency and weight computed by the LDAK software, respectively. Two cohorts do not have sample overlaps. Simulations under different parameters scenarios are repeated for 100 times.

Supplementary Table 4. Type I errors when two traits have different heritability values

	Type I error rate at significance level α=0.05			Empirical type I error rate at posterior probability cutoff 0.95	
Trait heritability	LOGODetect	ρ-HESS		coloc	gwas-pw
0.05	0.07	0		0	0.26
0.04	0.02	0		0	0.25
0.03	0.01	0		0	0.2
0.02	0.06	0		0	0.08
0.01	0.01	0		0	0.01

Type I error rates and empirical type I error rates denote the proportion of simulations that significant segments harboring local genetic correlation are identified under the null. Here we assume an infinitesimal model where per-SNP heritability for are all the SNPs are the same. Heritability for the first trait is set to vary from 0.2 to 0.01, heritability for the second trait is fixed at 0.02. Two cohorts do not have sample overlaps. Simulations under different parameters scenarios are repeated for 100 times.

Supplementary Table 5. Type I errors under an infinitesimal model with sample overlaps

	Type I error rate at significance level α=0.05			Empirical type I error rate at posterior probability cutoff 0.95	
Trait heritability	LOGODetect	ρ-HESS		coloc	gwas-pw
0.05	0.01	0		0	0.52
0.04	0.03	0		0.03	0.43
0.03	0.02	0		0.01	0.28
0.02	0.02	0		0	0.21
0.01	0.03	0		0	0.09

Type I error rates and empirical type I error rates denote the proportion of simulations that significant segments harboring local genetic correlation are identified under the null. Here we assume an infinitesimal model where per-SNP heritability for are all the SNPs are the same. Two cohorts share 50% overlapped samples. Simulations under different parameters scenarios are repeated for 100 times.

Supplementary Table 6. Type I errors for binary traits under a liability threshold model

	Type I error rate at significance level α=0.05			Empirical type I error rate at posterior probability cutoff 0.95	
Trait heritability	LOGODetect	ρ-HESS		coloc	gwas-pw
0.05	0.03	0		0	0.37
0.04	0.03	0		0	0.25
0.03	0.02	0		0	0.11
0.02	0.03	0		0	0.04
0.01	0.03	0		0	0.02

Type I error rates and empirical type I error rates denote the proportion of simulations that significant segments harboring local genetic correlation are identified under the null. Here we assume a liability threshold model that the disease status is determined by an unobserved continuous liability with sample prevalence being 0.5. The unobserved liability follows the infinitesimal model where per-SNP heritability for are all the SNPs are the same. Two cohorts do not have sample overlaps. Simulations under different parameters scenarios are repeated for 100 times.

Supplementary Table 7. Type I errors under an infinitesimal model with t-distributed effects

	Type I error rate at significance level α=0.05			Empirical type I error rate at posterior probability cutoff 0.95	
Trait heritability	LOGODetect	ρ-HESS		coloc	gwas-pw
0.05	0.07	0		0.01	0.43
0.04	0.05	0		0	0.34
0.03	0.02	0		0	0.17
0.02	0.05	0		0	0.07
0.01	0.02	0		0	0.01

Type I error rates and empirical type I error rates denote the proportion of simulations that significant segments harboring local genetic correlation are identified under the null. Here we assume an infinitesimal model with t-distributed effects, where effect sizes for both traits are proportional to the t-distribution with 10 degrees of freedom. Two cohorts do not have sample overlaps. Simulations under different parameters scenarios are repeated for 100 times.

Supplementary Table 8. Type I errors under a non-infinitesimal model with sparse effects

	Type I error rate at significance level α=0.05		Empirical type I error rate at posterior probability cutoff 0.95	
Trait heritability	LOGODetect	ρ-HESS	coloc	gwas-pw
0.05	0.03	0	0	0.46
0.04	0.01	0	0	0.38
0.03	0	0	0	0.26
0.02	0.02	0	0	0.08
0.01	0.03	0	0	0.02

Type I error rates and empirical type I error rates denote the proportion of simulations that significant segments harboring local genetic correlation are identified under the null. Here we assume a non-infinitesimal model with sparse effects. For the first trait we randomly sample 10000 causal variants from the first half of chr1, and we randomly sample 10000 causal variants from the second half for the second trait. Per-SNP heritability for all the causal variants are the same. Two cohorts do not have sample overlaps. Simulations under different parameters scenarios are repeated for 100 times.

Supplementary Table 9. Type I errors under an infinitesimal model

	Type I error rate at significance level α=0.05			Empirical type I error rate at posterior probability cutoff 0.95	
Trait heritability	LOGODetect	ρ-HESS		coloc	gwas-pw
0.2	0.08	0.59		0.2	0.52
0.1	0.07	0.01		0.05	0.51

Type I error rates and empirical type I error rates denote the proportion of simulations that significant segments harboring local genetic correlation are identified under the null. Here we assume an infinitesimal model where per-SNP heritability for all the SNPs are the same. Two cohorts do not have sample overlaps. Simulations under different parameters scenarios are repeated for 100 times.

Supplementary Table 10. Type I error rates under an alternative heritability enrichment model with varying heritability

Trait heritability	LOGODetect
0.05	0.06
0.04	0.06
0.03	0.05
0.02	0.01
0.01	0.01

Type I error rates denote the proportion of simulations that false positives at significance level α=0.05 are identified under an alternative heritability enrichment model. For each trait, we randomly choose N=5 segments, each contains L=1000 SNPs, as the signal regions. The heritability for the signal regions is set to be 30% trait heritability. The correlation of genetic effect size of two traits ρ is set to be 0.9. Each simulation setting is repeated for 100 times.

Supplementary Table 11. Type I error rates under an alternative heritability enrichment model with varying correlation

Correlation	LOGODetect
0.8	0.02
0.6	0.04
0.4	0.02
0.2	0.04

Type I error rates denote the proportion of simulations that false positives at significance level α=0.05 are identified under an alternative heritability enrichment model. For each trait, we randomly choose N=5 segments, each contains L=1000 SNPs, as the signal regions. The heritability for the signal regions is set to be 30% trait heritability. The trait heritability for two traits is set to be 0.03. Each simulation setting is repeated for 100 times.

Supplementary Table 12. Description of Sample size and reference for different datasets

Disease/Trait	Abbreviation	Reference	Sample size	Case	Control
Bipolar Disorder	BIP	Stahl, Eli A., et al. "Genome-wide association study identifies 30 loci associated with bipolar disorder." Nature genetics 51.5 (2019): 793.	51710	20352	31358
Schizophrenia	SCZ	Pardiñas, Antonio F., et al. "Common schizophrenia alleles are enriched in mutation-intolerant genes and in regions under strong background selection." Nature genetics 50.3 (2018): 381.	105318	40675	64643
Major Depressive Disorder	MDD	Wray, Naomi R., et al. "Genome-wide association analyses identify 44 risk variants and refine the genetic architecture of major depression." Nature genetics 50.5 (2018): 668.	173005	59851	113154
Neuroticism	NEU	Nagel, Mats, et al. "Meta-analysis of genome-wide association studies for neuroticism in 449,484 individuals identifies novel genetic loci and pathways." Nature genetics 50.7 (2018): 920-927.	390278	NA	NA
Attention-Deficit/Hyperactivity Disorder	ADHD	Demontis, Ditte, et al. "Discovery of the first genome-wide significant risk loci for attention deficit/hyperactivity disorder." Nature genetics 51.1 (2019): 63.	53293	19099	34194
Autism Spectrum Disorder	ASD	Grove, Jakob, et al. "Identification of common genetic risk variants for autism spectrum disorder." Nature genetics 51.3 (2019): 431.	46350	18381	27969
Intelligence	IQ	Savage, Jeanne E., et al. "Genome-wide association meta-analysis in 269,867 individuals identifies new genetic and functional links to intelligence." Nature genetics 50.7 (2018): 912-919.	269867	NA	NA

Supplementary Table 13. Proportion of genetic covariance identified by LOGODetect with varying θ

Trait 1	Trait 2	θ=0.4	θ=0.45	θ=0.5	θ=0.55	θ=0.6	θ=0.65	θ=0.7
BIP	SCZ	7.80%	8.80%	9.38%	11.12%	**11.63%**	10.59%	10.12%
BIP	MDD	0%	0%	1.74%	1.74%	**2.47%**	1.43%	1.43%
BIP	NEU	0%	0%	1.52%	0.57%	0.77%	2.47%	**2.49%**
BIP	ADHD	0%	0%	0%	0%	0%	0%	0%
BIP	ASD	0%	0%	0%	0%	0%	0%	0%
BIP	IQ	0%	0%	0%	-2.17%	**-12%**	0.82%	-5.44%
SCZ	MDD	4.25%	4.52%	6.12%	6.22%	6.93%	**7.45%**	6.38%
SCZ	NEU	2.74%	3.23%	5.19%	6.44%	9.06%	**14.13%**	13.89%
SCZ	ADHD	3.73%	3.41%	3.36%	3.36%	9.27%	**10.71%**	7.37%
SCZ	ASD	0%	0%	0%	0%	3.56%	3.57%	**3.94%**
SCZ	IQ	3.53%	4.31%	4.72%	4.38%	8.70%	7.08%	**9.38%**
MDD	NEU	3.35%	5.56%	6.77%	6.77%	**8.75%**	7.99%	8.33%
MDD	ADHD	1.66%	1.64%	**2.59%**	2.55%	2.50%	2.13%	1.29%
MDD	ASD	0.85%	1.08%	1.79%	**2.89%**	2.07%	1.53%	1.19%
MDD	IQ	0%	0%	0%	-1.39%	**-2.83%**	-2.81%	-2.81%
NEU	ADHD	0%	0%	0%	0%	3.78%	4.37%	**5.35%**
NEU	ASD	0%	0%	0%	-0.12%	**1.52%**	0%	1.06%
NEU	IQ	0%	0%	4.68%	5.62%	8.57%	9.12%	**10.35%**
ADHD	ASD	4.98%	5.64%	6.16%	**7.60%**	3.23%	2.60%	0.87%
ADHD	IQ	0.78%	1.29%	4.23%	6.43%	8.67%	**8.98%**	8.78%
ASD	IQ	0%	**3.57%**	3.55%	1.15%	1.96%	3.08%	2.68%

For each trait pair, proportion of genetic covariance identified by LOGODetect with different θ is calculated via stratified LDSC, and the largest absolute value of proportion of genetic covariance is highlighted in bold font.

Supplementary Table 14. Genetic correlation estimation using LDSC and local genetic correlation estimation using LOGODetect, ρ-HESS, coloc, and gwas-pw

Trait 1	Trait 2	Genetic correlation	s.e.	P	LOGODetect	ρ-HESS (full sample overlap)	ρ-HESS (approximate sample overlap)	coloc	gwas-pw
BIP	SCZ	0.68	0.034	9.14E-87	56	778	967	5	26
BIP	MDD	0.42	0.05	4.33E-17	6	0	0	0	4
BIP	NEU	0.14	0.03	9.99E-07	19	0	0	0	1
BIP	ADHD	0.18	0.054	8.00E-04	0	0	0	0	1
BIP	ASD	0.16	0.047	7.00E-04	0	0	0	1	1
BIP	IQ	-0.04	0.026	1.23E-01	19	1	1	3	6
SCZ	MDD	0.4	0.034	7.36E-33	14	93	36	0	7
SCZ	NEU	0.22	0.025	6.55E-18	38	131	118	5	19
SCZ	ADHD	0.2	0.041	6.55E-07	32	8	10	0	2
SCZ	ASD	0.26	0.043	2.32E-09	12	0	20	0	0
SCZ	IQ	-0.23	0.021	4.36E-28	53	304	237	10	24
MDD	NEU	0.78	0.059	6.38E-41	40	3	3	2	9
MDD	ADHD	0.61	0.076	1.72E-15	5	0	0	0	1
MDD	ASD	0.55	0.069	1.41E-15	7	0	0	0	1
MDD	IQ	-0.18	0.033	7.19E-08	3	0	0	2	5
NEU	ADHD	0.26	0.035	1.46E-13	12	0	0	1	3
NEU	ASD	0.18	0.037	9.05E-07	2	0	0	0	2
NEU	IQ	-0.19	0.021	7.68E-20	46	2	2	4	11
ADHD	ASD	0.42	0.067	4.46E-10	8	0	0	2	0
ADHD	IQ	-0.39	0.039	3.24E-23	30	1	1	0	2
ASD	IQ	0.23	0.036	1.47E-10	8	0	0	2	1

The column of Genetic correlation, s.e., and P represent the estimated genetic correlation, its corresponding standard error, and p value for each disease pair respectively using LDSC. Column of LOGODetect and ρ-HESS represent the numbers of detected significant segments using these approaches with FDR<0.05 respectively. Column of coloc and gwas-pw represent the numbers of detected significant segments using these approaches with PP (posterior probability) >0.95 respectively.

Supplementary Table 15. Approximated sample overlaps for different datasets

	BIP	SCZ	MDD	NEU	ADHD	ASD	IQ
BIP		40809	27933	0	1777	0	0
SCZ			29221	0	1413	0	0
MDD				29740	38247	36470	29740
NEU					0	0	195653
ADHD						35740	0
ASD							0
IQ							

We summarize the approximated sample overlap based on the cohort information detailed in the supplementary material of the GWAS references.

Supplementary Table 16. Sample size of replication cohorts from UKBB

Disease	Case	Control
Bipolar Disorder	1064	365476
Schizophrenia	571	365476

Supplementary Table 17. Genome segments showing correlations between BMI and height

Discovery study						
**Chr**	**Begin_pos (MB)**	**Stop_pos (MB)**	**Size (MB)**	**Sign**	**Pval**	**Qval**
**2**	**24.69**	**25.078**	**0.388**	**neg**	**0.00059988**	**0.011234117**
**2**	**25.079**	**25.174**	**0.095**	**neg**	**0.00019996**	**0.00509898**
**2**	**25.175**	**25.478**	**0.303**	**neg**	**0.00039992**	**0.009109289**
**2**	**219.235**	**219.744**	**0.509**	**neg**	**0.00019996**	**0.008158368**
**2**	**232.191**	**233.547**	**1.356**	**neg**	**0.00159968**	**0.022075585**
**3**	**72.383**	**72.516**	**0.133**	**neg**	**0.00179964**	**0.023282843**
**3**	**141.055**	**141.325**	**0.27**	**pos**	**0.00019996**	**0.013597281**
**5**	**122.64**	**122.791**	**0.151**	**pos**	**0.00559888**	**0.048523629**
**6**	**130.323**	**130.422**	**0.099**	**pos**	**0.00019996**	**0.01019796**
**6**	**142.634**	**142.864**	**0.23**	**neg**	**0.0009998**	**0.017246551**
**7**	**28.136**	**28.213**	**0.077**	**neg**	**0.00019996**	**0.00679864**
8	25.239	25.445	0.206	pos	0.0039992	0.041391722
**8**	**130.705**	**130.761**	**0.056**	**neg**	**0.00439912**	**0.041591682**
12	11.848	11.893	0.045	neg	0.00539892	0.048825018
12	66.342	66.373	0.031	pos	0.00019996	0.020395921
12	66.38	66.407	0.027	pos	0.00439912	0.043572238
13	27.956	28.121	0.165	pos	0.00159968	0.023652412
**13**	**32.977**	**33.382**	**0.405**	**neg**	**0.00239952**	**0.02614214**
14	92.402	92.558	0.156	neg	0.00179964	0.021913264
**15**	**89.383**	**89.436**	**0.053**	**neg**	**0.00139972**	**0.02228785**
**16**	**53.8**	**53.856**	**0.056**	**neg**	**0.00019996**	**0.005827406**
**18**	**57.733**	**57.998**	**0.265**	**pos**	**0.00019996**	**0.040791842**
**20**	**6.591**	**6.647**	**0.056**	**neg**	**0.00039992**	**0.00819836**
22	21.458	22.013	0.555	neg	0.00219956	0.025294941

Replication study						
**Chr**	**Begin_pos (MB)**	**Stop_pos (MB)**	**Size (MB)**	**Sign**	**Pval**	**Qval**
1	17.298	17.431	0.133	neg	0.00079984	0.006622675
1	92.68	93.471	0.791	neg	0.00919816	0.033051017
1	118.84	118.922	0.082	neg	0.00219956	0.013008827
2	0.621	0.656	0.035	pos	0.00059988	0.005644326
**2**	**24.68**	**25.071**	**0.391**	**neg**	**0.00019996**	**0.003726527**
**2**	**25.071**	**25.17**	**0.099**	**neg**	**0.00019996**	**0.002586983**
**2**	**25.28**	**25.488**	**0.208**	**neg**	**0.00019996**	**0.002942269**
2	44.354	44.892	0.538	neg	0.01159768	0.038779744
2	46.778	47.05	0.272	neg	0.00479904	0.02290451
**2**	**219.23**	**220.385**	**1.155**	**neg**	**0.00019996**	**0.004532427**
**2**	**232.306**	**233.142**	**0.836**	**neg**	**0.0009998**	**0.007665134**
3	53.1	53.179	0.079	neg	0.014597081	0.04300221
**3**	**72.382**	**72.4**	**0.018**	**neg**	**0.00039992**	**0.004357023**
3	135.625	136.669	1.044	neg	0.00039992	0.004139172
**3**	**141.028**	**141.129**	**0.101**	**pos**	**0.00879824**	**0.033913217**
3	185.781	185.858	0.077	neg	0.00779844	0.032111225
4	8.58	8.657	0.077	neg	0.00859828	0.034068658
4	17.784	18.052	0.268	neg	0.00019996	0.003153216
4	73.417	73.561	0.144	neg	0.013197361	0.041313476
4	145.254	145.423	0.169	neg	0.00839832	0.033916294
5	77.311	77.476	0.165	pos	0.00119976	0.008869655
5	88.772	88.876	0.104	neg	0.013797241	0.041202718
5	108.072	108.245	0.173	neg	0.016396721	0.046634829
**5**	**122.65**	**122.771**	**0.121**	**pos**	**0.00259948**	**0.014947011**
5	172.969	173.022	0.053	neg	0.00719856	0.030233953
5	176.51	176.537	0.027	neg	0.01179764	0.039022965
6	34.211	34.834	0.623	pos	0.01019796	0.035442092
6	105.349	105.481	0.132	neg	0.00859828	0.033596984
6	108.855	109.017	0.162	pos	0.00579884	0.026472966
6	126.648	127.089	0.441	neg	0.00019996	0.003415983
**6**	**130.334**	**130.404**	**0.07**	**pos**	**0.00019996**	**0.013597281**
**6**	**142.634**	**142.869**	**0.235**	**neg**	**0.00019996**	**0.00409918**
**7**	**28.14**	**28.208**	**0.068**	**neg**	**0.00019996**	**0.005827406**
7	148.64	148.653	0.013	neg	0.013597281	0.041169544
7	150.667	150.699	0.032	pos	0.017396521	0.048446007
8	24.09	24.162	0.072	neg	0.00879824	0.03272328
8	116.404	116.649	0.245	pos	0.0089982	0.032889974
**8**	**130.713**	**130.766**	**0.053**	**neg**	**0.00019996**	**0.00679864**
9	99.187	99.28	0.093	neg	0.00119976	0.008563804
10	12.894	12.952	0.058	neg	0.00219956	0.013797241
10	93.048	93.071	0.023	neg	0.00219956	0.013391439
10	99.691	100.053	0.362	neg	0.00019996	0.008158368
10	102.633	102.72	0.087	pos	0.00279944	0.015396921
10	104.308	104.406	0.098	neg	0.012797441	0.04125742

11	13.28	13.368	0.088	neg	0.00039992	0.004869614
11	65.633	65.666	0.033	neg	0.00359928	0.017996401
11	66.197	66.839	0.642	neg	0.00359928	0.01834755
11	115.016	115.103	0.087	neg	0.00719856	0.030850973
11	122.494	122.548	0.054	neg	0.00619876	0.027119576
11	128.485	128.507	0.022	neg	0.014797041	0.043207359
12	28.195	28.755	0.56	neg	0.0039992	0.019530978
12	94.078	94.215	0.137	neg	0.00179964	0.011641422
12	122.484	123.163	0.679	pos	0.00019996	0.01019796
**13**	**32.994**	**33.393**	**0.399**	**neg**	**0.00039992**	**0.00459908**
13	112.167	112.25	0.083	neg	0.00319936	0.017145289
14	23.75	23.813	0.063	pos	0.012797441	0.041688632
14	79.837	79.978	0.141	pos	0.00879824	0.033307624
14	103.853	104.071	0.218	neg	0.00319936	0.016716657
15	69.965	70.057	0.092	neg	0.00579884	0.025909712
15	74.213	74.516	0.303	neg	0.00159968	0.010681735
15	84.544	84.595	0.051	neg	0.017996401	0.049715057
**15**	**89.34**	**89.412**	**0.072**	**neg**	**0.00019996**	**0.00509898**
15	99.2	99.226	0.026	neg	0.013397321	0.041135435
16	2.085	2.27	0.185	pos	0.00579884	0.027061254
16	28.289	29.007	0.718	pos	0.013397321	0.041531694
16	29.921	30.119	0.198	pos	0.00019996	0.020395921
**16**	**53.8**	**53.847**	**0.047**	**neg**	**0.00059988**	**0.005173965**
16	69.534	70.503	0.969	neg	0.012997401	0.04128586
17	7.347	7.439	0.092	neg	0.00979804	0.034619743
17	21.243	21.287	0.044	neg	0.00079984	0.006367957
17	61.612	61.774	0.162	neg	0.01019796	0.034870445
18	21.069	21.168	0.099	pos	0.00159968	0.011037792
**18**	**57.732**	**58.01**	**0.278**	**pos**	**0.00019996**	**0.040791842**
19	4.028	4.135	0.107	neg	0.00059988	0.00539892
19	4.92	4.972	0.052	neg	0.01159768	0.039211205
19	30.984	31.057	0.073	neg	0.00039992	0.003942069
**20**	**6.409**	**6.581**	**0.172**	**neg**	**0.00259948**	**0.014613294**
20	6.582	6.646	0.064	neg	0.00019996	0.002746117
21	41.387	41.464	0.077	neg	0.014997001	0.043215041
22	45.688	45.864	0.176	neg	0.017196561	0.048282651

These two tables show all detected regions between BMI and height in both discovery and replication studies. The location, scan statistic sign, p-value, and q-value for each detected region are provided. The tables are organized following the order of chromosome and the beginning position of the detected region. The region identified in both the discovery and replication studies are highlighted in bold font.

Supplementary Table 18. Overlaps between 66 GenoSkylinePlus annotations and 6 generic annotations

ID	Tissue_type	Cell_type	Coding	Enhancer	Intron	Promoter	UTR_3	UTR_5
E062	Blood	Mononuclear cells from peripheral blood	0.42	0.56	0.73	0.52	0.12	0.34
E034	Blood	T cells CD3+	0.19	0.31	0.51	0.23	0.07	0.12
E045	Blood	T cells effector/memory CD4+ CD25int CD127+	0.25	0.54	0.62	0.34	0.08	0.19
E044	Blood	T regulatory cells CD4+ CD25+ CD127-	0.19	0.41	0.56	0.27	0.07	0.13
E043	Blood	T helper cells CD4+ CD25-	0.2	0.4	0.57	0.27	0.07	0.14
E039	Blood	T helper naive cells CD4+ CD25- CD45RA+	0.21	0.44	0.59	0.29	0.07	0.15
E041	Blood	T helper cells PMA-I stimulated CD4+ CD25- IL17-	0.19	0.37	0.58	0.24	0.07	0.12
E042	Blood	T helper 17 cells PMA-I stimulated CD4+ CD25- IL17+	0.2	0.44	0.57	0.29	0.07	0.15
E040	Blood	T helper memory cells CD4+ CD25- CD45RO+	0.24	0.55	0.62	0.35	0.08	0.19
E037	Blood	T helper memory cells from peripheral blood CD4+	0.23	0.56	0.62	0.34	0.07	0.18
E048	Blood	T memory cells CD8+	0.24	0.56	0.61	0.36	0.08	0.2
E038	Blood	T helper naive cells CD4+	0.22	0.49	0.57	0.31	0.07	0.17
E047	Blood	T naive cells CD8+	0.2	0.45	0.54	0.28	0.06	0.15
E029&E124	Blood	Monocytes CD14+	0.22	0.4	0.6	0.28	0.08	0.14
E051	Blood	Hematopoietic stem cells G-CSF-mobilized CD34+	0.21	0.43	0.53	0.33	0.07	0.16
E032	Blood	B cells CD19+	0.23	0.46	0.54	0.34	0.08	0.16
E046	Blood	Natural killer cells CD56+	0.24	0.48	0.56	0.36	0.08	0.18
E116	Blood	GM12878 lymphoblastoid cells	0.23	0.58	0.65	0.32	0.07	0.18
E112	Thymus	Thymus	0.25	0.55	0.58	0.38	0.08	0.2
E113	Spleen	Spleen	0.28	0.55	0.66	0.36	0.09	0.2
E071	Brain	Brain hippocampus middle	0.27	0.48	0.7	0.34	0.09	0.19
E074	Brain	Brain substantia nigra	0.27	0.46	0.72	0.35	0.09	0.2
E068	Brain	Brain anterior caudate	0.24	0.42	0.7	0.3	0.08	0.16
E069	Brain	Brain cingulate gyrus	0.29	0.46	0.72	0.35	0.09	0.2
E072	Brain	Brain inferior temporal lobe	0.31	0.45	0.73	0.36	0.1	0.21
E067	Brain	Brain angular gyrus	0.29	0.47	0.73	0.37	0.09	0.2
E073	Brain	Brain dorsolateral prefrontal cortex	0.31	0.49	0.72	0.39	0.1	0.22
E125	Brain	NH-A astrocytes	0.23	0.56	0.63	0.33	0.07	0.18
E096	Lung	Lung	0.27	0.55	0.65	0.36	0.09	0.21
E128	Lung	NHLF lung fibroblast primary cells	0.31	0.58	0.67	0.41	0.09	0.26
E065	Vascular	Aorta	0.37	0.64	0.66	0.54	0.1	0.34
E104	Heart	Right atrium	0.26	0.55	0.67	0.38	0.08	0.21
E095	Heart	Left ventricle	0.29	0.56	0.7	0.39	0.09	0.23
E105	Heart	Right ventricle	0.32	0.54	0.69	0.41	0.1	0.24
E052	Muscle	Muscle satellite cultured cells	0.24	0.56	0.62	0.35	0.07	0.19
E100	Muscle	Psoas muscle	0.4	0.59	0.69	0.54	0.11	0.35
E107&E108	Muscle	Skeletal muscle	0.21	0.41	0.58	0.27	0.07	0.13
E120	Muscle	HSMM skeletal muscle myoblasts cells	0.19	0.42	0.58	0.25	0.06	0.15
E121	Muscle	HSMM cell derived skeletal muscle myotubes cells	0.22	0.46	0.62	0.28	0.07	0.17
E111	GI	Stomach smooth muscle	0.29	0.51	0.69	0.38	0.09	0.22
E078	GI	Duodenum smooth muscle	0.33	0.62	0.68	0.49	0.09	0.28
E076	GI	Colon smooth muscle	0.27	0.52	0.69	0.39	0.08	0.21
E103	GI	Rectal smooth muscle	0.29	0.52	0.69	0.39	0.09	0.23
E079	GI	Esophagus	0.32	0.6	0.66	0.44	0.09	0.27
E094	GI	Gastric	0.27	0.53	0.62	0.36	0.08	0.23
E110	GI	Stomach mucosa	0.26	0.61	0.63	0.39	0.08	0.21
E077	GI	Duodenum mucosa	0.28	0.6	0.66	0.39	0.09	0.21
E109	GI	Small intestine	0.31	0.61	0.67	0.46	0.09	0.26
E106	GI	Sigmoid colon	0.37	0.62	0.68	0.49	0.11	0.3
E075	GI	Colonic mucosa	0.34	0.61	0.66	0.46	0.1	0.27
E101&E102	GI	Rectal mucosa	0.26	0.56	0.65	0.38	0.08	0.2
E098	Pancreas	Pancreas	0.26	0.48	0.59	0.34	0.08	0.21
E087	Pancreas	Pancreatic islets	0.51	0.63	0.71	0.74	0.12	0.48
E066	Liver	Liver	0.26	0.52	0.68	0.35	0.08	0.19
E063	Fat	Adipose nuclei	0.23	0.52	0.66	0.34	0.08	0.18
E025	Fat	Adipose derived mesenchymal stem cell cultured cells	0.21	0.51	0.62	0.3	0.07	0.16
E023	Fat	Mesenchymal stem cell derived adipocyte cultured cells	0.22	0.51	0.62	0.31	0.07	0.17
E049	Bone/connective	Mesenchymal stem cell derived chondrocyte cultured cells	0.21	0.47	0.65	0.29	0.07	0.15
E026	Bone/connective	Bone marrow derived cultured mesenchymal stem cells	0.2	0.49	0.62	0.28	0.06	0.15
E129	Bone/connective	Osteoblast primary cells	0.22	0.52	0.63	0.3	0.07	0.17
E126	Skin	NHDF-Ad adult dermal fibroblast primary cells	0.2	0.42	0.58	0.28	0.06	0.16
E127	Skin	NHEK-epidermal keratinocyteprimary cells	0.24	0.5	0.62	0.34	0.07	0.19
E027	Breast	Breast myoepithelial primary cells	0.27	0.59	0.65	0.38	0.08	0.22
E028	Breast	Breast variant human mammary epithelial cells (vHMEC)	0.29	0.63	0.66	0.43	0.08	0.25
E119	Breast	HMEC mammary epithelial primary cells	0.25	0.53	0.6	0.37	0.07	0.21
E097	Ovary	Ovary	0.3	0.49	0.63	0.37	0.1	0.22

Each row represents the proportion of cell type specific annotation overlapped with six generic annotations. The two significantly enriched brain tissue types, i.e. cingulate gyrus and angular gyrus, are highlighted with yellow background.

Supplementary Table 19. Gene Ontology analysis

Category	GeneSet	N_genes	N_overlap	p	adjP
GO_bp	GO_RNA_METABOLIC_PROCESS	1542	85	5.36E-13	3.94E-09
GO_bp	GO_REGULATION_OF_CELL_DIFFERENTIATION	1844	86	1.85E-09	6.81E-06
GO_bp	GO_APOPTOTIC_PROCESS	1956	89	3.10E-09	7.59E-06
GO_bp	GO_HISTONE_CITRULLINATION	5	5	7.35E-09	1.35E-05
GO_bp	GO_REGULATION_OF_CELL_DEATH	1697	79	9.66E-09	1.42E-05
GO_bp	GO_NEGATIVE_REGULATION_OF_CELL_DEATH	1003	53	6.09E-08	7.46E-05
GO_bp	GO_NEGATIVE_REGULATION_OF_BIOSYNTHETIC_PROCESS	1612	71	4.56E-07	0.00047919
GO_bp	GO_ORGANONITROGEN_COMPOUND_BIOSYNTHETIC_PROCESS	1814	77	6.13E-07	0.00056274
GO_bp	GO_MRNA_METABOLIC_PROCESS	560	34	7.16E-07	0.00058471
GO_bp	GO_RNA_SPLICING	456	29	1.87E-06	0.00137558
GO_bp	GO_REGULATION_OF_CELL_POPULATION_PROLIFERATION	1684	71	2.18E-06	0.0014546
GO_bp	GO_PRODUCTION_OF_SMALL_RNA_INVOLVED_IN_GENE_SILENCING_BY_RNA	52	9	3.31E-06	0.00186612
GO_bp	GO_ORGANIC_CYCLIC_COMPOUND_CATABOLIC_PROCESS	601	34	3.40E-06	0.00186612
GO_bp	GO_NEGATIVE_REGULATION_OF_RNA_BIOSYNTHETIC_PROCESS	1238	56	3.55E-06	0.00186612
GO_bp	GO_MACROMOLECULE_CATABOLIC_PROCESS	1366	60	3.98E-06	0.00194808
GO_bp	GO_NEGATIVE_REGULATION_OF_DEVELOPMENTAL_PROCESS	1013	48	5.31E-06	0.00243856
GO_bp	GO_CELLULAR_MACROMOLECULE_CATABOLIC_PROCESS	1139	52	6.15E-06	0.00266026
GO_bp	GO_CENTRAL_NERVOUS_SYSTEM_DEVELOPMENT	972	46	8.59E-06	0.00332645
GO_bp	GO_RNA_CATABOLIC_PROCESS	391	25	8.60E-06	0.00332645
GO_bp	GO_RNA_SPLICING_VIA_TRANSESTERIFICATION_REACTIONS	369	24	9.75E-06	0.00358329
GO_bp	GO_NEGATIVE_REGULATION_OF_CELL_DIFFERENTIATION	733	37	1.66E-05	0.00579878
GO_bp	GO_PHOSPHATIDYLSERINE_METABOLIC_PROCESS	25	6	2.08E-05	0.00696519
GO_bp	GO_PEPTIDYL_ARGININE_MODIFICATION	27	6	3.35E-05	0.01069499
GO_bp	GO_PHOSPHATIDYLINOSITOL_ACYL_CHAIN_REMODELING	17	5	3.59E-05	0.01099383
GO_bp	GO_PHOSPHATIDYLGLYCEROL_ACYL_CHAIN_REMODELING	18	5	4.87E-05	0.01432863
GO_bp	GO_POSITIVE_REGULATION_OF_GENE_EXPRESSION	1955	74	5.18E-05	0.01465118
GO_bp	GO_ORGANOPHOSPHATE_BIOSYNTHETIC_PROCESS	553	29	6.83E-05	0.01860578
GO_bp	GO_RNA_DESTABILIZATION	31	6	7.68E-05	0.01946362
GO_bp	GO_ARACHIDONIC_ACID_SECRETION	31	6	7.68E-05	0.01946362
GO_bp	GO_POSITIVE_REGULATION_OF_AUTOPHAGY_OF_MITOCHONDRION	20	5	8.48E-05	0.02077369
GO_bp	GO_FATTY_ACID_TRANSPORT	96	10	9.45E-05	0.02239825
GO_bp	GO_NEUROGENESIS	1594	62	0.00010132	0.02288209
GO_bp	GO_NUCLEOSIDE_PHOSPHATE_BIOSYNTHETIC_PROCESS	273	18	0.00010274	0.02288209
GO_bp	GO_PHOSPHATIDYLSERINE_ACYL_CHAIN_REMODELING	21	5	0.00010912	0.02354522
GO_bp	GO_FATTY_ACID_DERIVATIVE_TRANSPORT	47	7	0.00011212	0.02354522
GO_bp	GO_NEURAL_TUBE_DEVELOPMENT	160	13	0.00012058	0.02461806
GO_bp	GO_REGULATION_OF_LAMELLIPODIUM_ORGANIZATION	48	7	0.0001286	0.02554652
GO_bp	GO_PEPTIDYL_AMINO_ACID_MODIFICATION	1219	50	0.00013829	0.02674872
GO_bp	GO_GLIAL_CELL_DIFFERENTIATION	209	15	0.00015118	0.02849071
GO_bp	GO_INTRINSIC_APOPTOTIC_SIGNALING_PATHWAY	284	18	0.00016824	0.03091383
GO_bp	GO_REGULATION_OF_CELL_CYCLE	1201	49	0.00018208	0.03189187
GO_bp	GO_REGULATION_OF_LAMELLIPODIUM_ASSEMBLY	36	6	0.00018371	0.03189187
GO_bp	GO_HEAD_DEVELOPMENT	764	35	0.00018658	0.03189187
GO_bp	GO_LONG_CHAIN_FATTY_ACID_TRANSPORT	69	8	0.00022321	0.03728549
GO_bp	GO_NON_CANONICAL_WNT_SIGNALING_PATHWAY	149	12	0.0002362	0.03857865
GO_bp	GO_RIBONUCLEOPROTEIN_COMPLEX_BIOGENESIS	483	25	0.00025719	0.03951283
GO_bp	GO_PURINE_CONTAINING_COMPOUND_BIOSYNTHETIC_PROCESS	196	14	0.00026056	0.03951283
GO_bp	GO_SMALL_MOLECULE_METABOLIC_PROCESS	1685	63	0.00026129	0.03951283
GO_bp	GO_PHOSPHATIDYLGLYCEROL_METABOLIC_PROCESS	25	5	0.00026342	0.03951283
GO_bp	GO_REGULATION_OF_INTRACELLULAR_SIGNAL_TRANSDUCTION	1824	67	0.00027347	0.04019987
GO_bp	GO_PHOSPHATIDYLETHANOLAMINE_ACYL_CHAIN_REMODELING	26	5	0.00031981	0.04609079
GO_bp	GO_PRE_MIRNA_PROCESSING	15	4	0.00034574	0.04886891
GO_cc	GO_NUCLEOLUS	1342	58	9.30E-06	0.00930896
GO_cc	GO_CATALYTIC_COMPLEX	1351	57	2.20E-05	0.01100483
GO_cc	GO_SM_LIKE_PROTEIN_FAMILY_COMPLEX	109	11	5.73E-05	0.01669703
GO_cc	GO_NUCLEAR_BODY	769	36	9.95E-05	0.01669703
GO_cc	GO_CONNEXIN_COMPLEX	21	5	0.00010912	0.01669703
GO_cc	GO_NUCLEOPLASM_PART	1113	47	0.00011236	0.01669703
GO_cc	GO_INTRINSIC_COMPONENT_OF_ORGANELLE_MEMBRANE	378	22	0.00011676	0.01669703
GO_cc	GO_CELL_PROJECTION_PART	1438	56	0.00021191	0.02489839
GO_cc	GO_NEURON_PART	1709	64	0.00022386	0.02489839
GO_cc	GO_RIBONUCLEOPROTEIN_COMPLEX	1361	53	0.00031141	0.0311726
GO_cc	GO_CELL_BODY	557	27	0.00041695	0.03353061
GO_cc	GO_UBIQUITIN_LIGASE_COMPLEX	281	17	0.00043195	0.03353061
GO_cc	GO_CAJAL_BODY	76	8	0.00043546	0.03353061
GO_cc	GO_NEURON_PROJECTION	1301	50	0.00061146	0.04371963
GO_mf	GO_PROTEIN_ARGININE_DEIMINASE_ACTIVITY	5	5	7.35E-09	1.21E-05
GO_mf	GO_HYDROLASE_ACTIVITY_ACTING_ON_CARBON_NITROGEN_BUT_NOT_PEPTIDE_BONDS_IN_LINEAR_AMIDINES	11	6	7.22E-08	5.94E-05
GO_mf	GO_TUMOR_NECROSIS_FACTOR_ACTIVATED_RECEPTOR_ACTIVITY	9	5	8.56E-07	0.00046514
GO_mf	GO_CALCIUM_DEPENDENT_PHOSPHOLIPASE_A2_ACTIVITY	16	6	1.13E-06	0.00046514
GO_mf	GO_DEATH_RECEPTOR_ACTIVITY	11	5	3.02E-06	0.0009925
GO_mf	GO_CARBOXYLIC_ESTER_HYDROLASE_ACTIVITY	133	13	1.76E-05	0.00481962
GO_mf	GO_RNA_BINDING	1875	73	2.38E-05	0.00558161
GO_mf	GO_MIRNA_BINDING	29	6	5.16E-05	0.01060675
GO_mf	GO_TRANSCRIPTION_FACTOR_BINDING	639	32	7.02E-05	0.01158939
GO_mf	GO_PHOSPHOLIPASE_A2_ACTIVITY	31	6	7.68E-05	0.01158939
GO_mf	GO_TRANSCRIPTION_COREGULATOR_ACTIVITY	557	29	7.75E-05	0.01158939
GO_mf	GO_PHOSPHOLIPASE_A2_ACTIVITY_CONSUMING_1_2_DIPALMITOYLPHOSPHATIDYLCHOLINE_	21	5	0.00010912	0.01495898
GO_mf	GO_HYDROLASE_ACTIVITY_ACTING_ON_ESTER_BONDS	723	34	0.00014131	0.01788101
GO_mf	GO_REGULATORY_RNA_BINDING	37	6	0.00021491	0.02525197
GO_mf	GO_ALDO_KETO_REDUCTASE_NADP_ACTIVITY	26	5	0.00031981	0.0350729
GO_mf	GO_SEQUENCE_SPECIFIC_DNA_BINDING	1114	45	0.00040747	0.0418934
GO_mf	GO_DNA_BINDING_TRANSCRIPTION_FACTOR_ACTIVITY	1691	62	0.00048243	0.04668193

968 prioritized genes show significant enrichment in three gene ontology domains: GO biological process (GO_bp), GO cellular component (GO_cc), GO molecular function (GO_mf).

Supplementary Table 20. Putative target genes for five hub regions identified in at least seven pair-wise analyses

Locus	LOGODetect	ρ-HESS	coloc	gwas-pw	Putative target genes
Chr11: 112.742-113.453MB	BIP-SCZ, BIP-NEU, BIP-IQ, SCZ-MDD, SCZ-NEU, SCZ-IQ, MDD-NEU, NEU-ADHD, NEU-IQ	BIP-SCZ, SCZ-NEU, SCZ-IQ		SCZ-NEU	*DRD2, NCAM1*
Chr11: 133.793-134.281MB	BIP-SCZ, SCZ-MDD, SCZ_NEU, SCZ_ADHD, SCZ-IQ, NEU-IQ, ADHD-IQ	BIP-SCZ, SCZ-NEU, SCZ-IQ		SCZ-NEU, NEU-IQ	*IGSF9B*
Chr14: 29.500-30.194MB	BIP-SCZ, BIP-IQ, SCZ-NEU, SCZ-ADHD, SCZ-IQ, NEU-IQ, ADHD-IQ	BIP-SCZ			*PRKD1, FOXG1*
Chr3: 71.434-71.692MB	SCZ-NEU, SCZ-ADHD, SCZ-ASD, SCZ-IQ, NEU-IQ, ADHD-IQ, ASD-IQ	BIP-SCZ, SCZ-NEU, SCZ-IQ	SCZ-IQ	SCZ-IQ	*FOXP1*
Chr10: 106.385-106.835MB	SCZ-NEU, SCZ-ADHD, MDD-NEU, MDD-ADHD, NEU-ADHD, ADHD-ASD, ADHD-IQ	BIP-SCZ, SCZ-MDD, SCZ-IQ		SCZ-ADHD	*SORCS3*

Here we listed out the five hub regions with significant local genetic correlation in at least seven trait pairs identified by LOGODetect. The second to fifth columns show the trait pairs with significant genetic correlation at the corresponding locus identified by LOGODetect, ρ-HESS, coloc, and gwas-pw, respectively.

Supplementary References

1. Jeng, X.J., Cai, T.T. & Li, H. Optimal sparse segment identification with application in copy number variation analysis. *Journal of the American Statistical Association* **105**, 1156-1166 (2010).
2. Li, Z., Liu, Y. & Lin, X. Simultaneous Detection of Signal Regions Using Quadratic Scan Statistics With Applications in Whole Genome Association Studies. *arXiv preprint arXiv:1710.05021* (2017).
3. Bulik-Sullivan, B. *et al.* An atlas of genetic correlations across human diseases and traits. *Nature genetics* **47**, 1236 (2015).
4. Consortium, G.P. A global reference for human genetic variation. *Nature* **526**, 68-74 (2015).
5. Su, Z., Marchini, J. & Donnelly, P. HAPGEN2: simulation of multiple disease SNPs. *Bioinformatics* **27**, 2304-2305 (2011).
6. Shi, H., Mancuso, N., Spendlove, S. & Pasaniuc, B. Local genetic correlation gives insights into the shared genetic architecture of complex traits. *The American Journal of Human Genetics* **101**, 737-751 (2017).
7. Speed, D., Cai, N., Johnson, M.R., Nejentsev, S. & Balding, D.J. Reevaluation of SNP heritability in complex human traits. *Nature genetics* **49**, 986-992 (2017).
8. Berisa, T. & Pickrell, J.K. Approximately independent linkage disequilibrium blocks in human populations. *Bioinformatics* **32**, 283 (2016).
9. Finucane, H.K. *et al.* Partitioning heritability by functional annotation using genome-wide association summary statistics. *Nature genetics* **47**, 1228 (2015).
10. Lu, Q. *et al.* A Powerful Approach to Estimating Annotation-Stratified Genetic Covariance via GWAS Summary Statistics. *Am J Hum Genet* **101**, 939-964 (2017).
11. Zhao, Z. *et al.* Fine-tuning Polygenic Risk Scores with GWAS Summary Statistics. *bioRxiv*, 810713 (2019).
12. Wood, A.R. *et al.* Defining the role of common variation in the genomic and biological architecture of adult human height. *Nature genetics* **46**, 1173-1186 (2014).
13. Locke, A.E. *et al.* Genetic studies of body mass index yield new insights for obesity biology. *Nature* **518**, 197-206 (2015).
