# Supplementary material for: Detecting local genetic correlations with scan statistics
Source: Nat Commun. 2021 Apr 1;12:2033. doi: 10.1038/s41467-021-22334-6 (PMC8016883; doi:10.1038/s41467-021-22334-6)
Supplement: Supplementary file 3 — Descriptions of Additional Supplementary Files [file 41467_2021_22334_MOESM3_ESM.docx]

Descriptions of Supplementary Files

**Supplementary Data 1**

**Description:** Genome segments identified by LOGODetect.

**Legend of Supplementary Data 2**

**Description: Identified** segments using LOGODetect.
