# Supplementary material for: Detecting local genetic correlations with scan statistics
Source: Nat Commun. 2021 Apr 1;12:2033. doi: 10.1038/s41467-021-22334-6 (PMC8016883; doi:10.1038/s41467-021-22334-6)
Supplement: Supplementary file 6 — Reporting Summary [file 41467_2021_22334_MOESM6_ESM.pdf]

nature research

Corresponding author(s): Lin Hou

Last updated by author(s): Feb 24, 2021

# Reporting Summary

Nature Research wishes to improve the reproducibility of the work that we publish. This form provides structure for consistency and transparency in reporting. For further information on Nature Research policies, see Authors & Referees and the Editorial Policy Checklist .

## Statistics

For all statistical analyses, confirm that the following items are present in the figure legend, table legend, main text, or Methods section.

| n/a | Confirmed | |
|---|---|---|
| ☐ | ☒ | The exact sample size (*n*) for each experimental group/condition, given as a discrete number and unit of measurement |
| ☐ | ☒ | A statement on whether measurements were taken from distinct samples or whether the same sample was measured repeatedly |
| ☐ | ☒ | The statistical test(s) used AND whether they are one- or two-sided<br>*Only common tests should be described solely by name; describe more complex techniques in the Methods section.* |
| ☐ | ☒ | A description of all covariates tested |
| ☐ | ☒ | A description of any assumptions or corrections, such as tests of normality and adjustment for multiple comparisons |
| ☐ | ☒ | A full description of the statistical parameters including central tendency (e.g. means) or other basic estimates (e.g. regression coefficient) AND variation (e.g. standard deviation) or associated estimates of uncertainty (e.g. confidence intervals) |
| ☐ | ☒ | For null hypothesis testing, the test statistic (e.g. *F*, *t*, *r*) with confidence intervals, effect sizes, degrees of freedom and *P* value noted<br>*Give P values as exact values whenever suitable.* |
| ☐ | ☒ | For Bayesian analysis, information on the choice of priors and Markov chain Monte Carlo settings |
| ☐ | ☒ | For hierarchical and complex designs, identification of the appropriate level for tests and full reporting of outcomes |
| ☐ | ☒ | Estimates of effect sizes (e.g. Cohen's *d*, Pearson's *r*), indicating how they were calculated |

*Our web collection on statistics for biologists contains articles on many of the points above.*

## Software and code

Policy information about availability of computer code

| | |
|---|---|
| Data collection | No software was used for data collection. |
| Data analysis | We have developed the LOGODetect software which is publicly available at https://github.com/ghm17/LOGODetect (https://doi.org/10.5281/zenodo.4559388).<br>We have used FUMA v1.3.6, ρ-HESS v0.5.3-beta , coloc v3.2-1, gwas-pw v0.21, LDSC v1.0.1, LDAK v5.1, HAPGEN2 v2.2.0, and ldetect in the data analysis process. The link to these softwares are provided in the manuscript. |

For manuscripts utilizing custom algorithms or software that are central to the research but not yet described in published literature, software must be made available to editors/reviewers. We strongly encourage code deposition in a community repository (e.g. GitHub). See the Nature Research guidelines for submitting code & software for further information.

## Data

Policy information about availability of data

All manuscripts must include a data availability statement. This statement should provide the following information, where applicable:

- Accession codes, unique identifiers, or web links for publicly available datasets
- A list of figures that have associated raw data
- A description of any restrictions on data availability

Summary statistics data of five psychiatric disorder were downloaded on the PGC website, http://www.med.unc.edu/pgc/downloads; Summary statistics data of neuroticism and intelligence were downloaded at the website of the Department of Complex Trait Genetics, CNCR, https://ctg.cncr.nl/software/summary_statistics; Summary statistics data of body-mass index and height were downloaded on the GIANT consortium website http://portals.broadinstitute.org/collaboration/giant/index.php/GIANT_consortium_data_files; Summary statistics for bipolar disorder, schizophrenia, body-mass index, and height in the replication cohort were downloaded from UK Biobank repository, http://www.nealelab.is/uk-biobank; phase 3 of the 1000 Genomes Project ftp://ftp.1000genomes.ebi.ac.uk/vol1/ftp/release/20130502/; 66 GenoSkyline-Plus cell-type specific functional annotations, http://genocanyon.med.yale.edu/GenoSkyline.

# Field-specific reporting

Please select the one below that is the best fit for your research. If you are not sure, read the appropriate sections before making your selection.

- [x] Life sciences
- [ ] Behavioural & social sciences
- [ ] Ecological, evolutionary & environmental sciences

For a reference copy of the document with all sections, see nature.com/documents/nr-reporting-summary-flat.pdf

# Life sciences study design

All studies must disclose on these points even when the disclosure is negative.

| | |
|---|---|
| Sample size | Previous studies have suggested strong genetic correlations among neuropsychiatric traits. Therefore, we applied LOGODetect to seven latest and well-powered GWAS summary statistics of neuropsychiatric traits. The sample size was determined by each GWAS study. |
| Data exclusions | None. |
| Replication | We used GWAS summary statistics of Bipolar Disorder (Stahl, E.A. et al. [2019]) and Schizophrenia (Pardiñas, A.F. et al. [2018]) to search regions enriched for local genetic correlations. We successfully replicated significant aggregated genetic covariance in the independent cohorts in the UK BioBank released in 2018. |
| Randomization | Randomization is irrelevant to our study as GWAS summary statistics are based on observational studies. |
| Blinding | Blinding is irrelevant to our study as GWAS summary statistics are based on observational studies. |

# Reporting for specific materials, systems and methods

We require information from authors about some types of materials, experimental systems and methods used in many studies. Here, indicate whether each material, system or method listed is relevant to your study. If you are not sure if a list item applies to your research, read the appropriate section before selecting a response.

## Materials & experimental systems

| n/a | Involved in the study | |
|---|---|---|
| x | | Antibodies |
| x | | Eukaryotic cell lines |
| x | | Palaeontology |
| x | | Animals and other organisms |
| x | | Human research participants |
| x | | Clinical data |

## Methods

| n/a | Involved in the study | |
|---|---|---|
| x | | ChIP-seq |
| x | | Flow cytometry |
| x | | MRI-based neuroimaging |
